# Comparative analysis of the impact of interferon regulatory factors on basal and induced interferon and interferon stimulated gene expression in human and canine keratinocytes

**DOI:** 10.3389/fimmu.2026.1810139

**Published:** 2026-05-15

**Authors:** Sarah Quinlan, Susan May, Ryan Weeks, Jennifer Luff

**Affiliations:** North Carolina State University, Department of Population Health and Pathobiology, Raleigh, NC, United States

**Keywords:** dog, interferon, interferon regulatory factor, interferon stimulated gene, keratinocyte

## Abstract

**Introduction:**

Keratinocytes are key barrier cells able to mount a robust interferon (IFN) antiviral response to defend against infection in the skin. Similar to humans, dogs spontaneously develop skin disorders associated with dysregulation of IFN immunity and can be used as a large animal model to investigate these diseases. One of the critical factors driving IFN regulation are interferon regulatory factors (IRFs). IRFs are crucial in upregulating the antiviral type I or type III IFNs, which then subsequently upregulate hundreds of antiviral effector proteins called interferon stimulated genes (ISGs).

**Methods:**

We sought to comparatively analyze how IRF1, 3 and 7 regulate type I and type III IFNs and ISGs using canine and human keratinocyte cultures. To this aim, we stimulated keratinocytes *in vitro* with dsDNA and dsRNA ligands and analyzed upregulation of IFNs and ISGs using real time quantitative PCR (RT-qPCR). Knockout of IRF3 and IRF1 and knockdown of IRF7 was performed on dog and human keratinocytes using CRISPR-Cas9 and shRNA methods, respectively. Knockdown or knockout was confirmed using RT-qPCR and/or western blots.

**Results:**

We demonstrated that compared to canine keratinocytes, human keratinocytes express higher basal type I IFN-β and ISGs, induce higher levels of type III IFNs upon stimulation, and overall, express higher IFN and ISG copies. We further showed that IRF regulation of induced IFNs, particularly IRF1, can be species-specific and/or stimulus-specific, and putatively that non-IRF mechanisms likely mediate basal expression of type I IFNs in human keratinocytes and type III IFNs in canine keratinocytes. While both IRF3 and IRF7 are critical for type I and III IFN induction following activation of cytosolic dsDNA and dsRNA receptors, neither can account for differences in the induced type I versus III IFN expression between dog and human, suggesting non-IRF1, 3, or 7 mechanisms underly this regulation.

**Conclusions:**

These studies provide support for use of the dog as a model to discern mechanisms underlying high basal type I and ISGs in human keratinocytes, how high basal ISGs can modulate the antiviral response, and uncover non-IRF mediated mechanisms regulating the type I versus type III response in keratinocytes.

## 1 Introduction

Interferons (IFN) are a family of cellular proteins, called cytokines, that were discovered to have antiviral function and aptly named due to their ability to “interfere” with viral replication (1). They have since been shown to be crucial in innate immunity, by controlling pathogen infection through induction of molecular countermeasures in the cells. There are three types of IFNs in mammals. Type II, which consists of IFN-γ, is expressed by immune cells and has primarily immunomodulatory and proinflammatory functions, distinct from type I and type III, which are both more antiviral in nature (2). In humans, there are 17 subtypes of type I IFN, including 13 types of IFN-α, in addition to IFN-β, IFN-ε, IFN-κ, and IFN-ω. The type III family consists of IFN-λ1 (also known as IL-29), IFN-λ2 (IL-28A), IFN-λ3 (IL-28B), and recently, IFN-λ4 (1). Induction of both type I and type III IFNs by pathogen infection is initiated when cellular pattern recognition receptors (PRRs) such as surface and endosomal toll-like receptors (TLRs), or cytosolic RNA and DNA sensors such as RIG-I and MDA5 (dsRNA sensors) and IFI16 and cGAS (dsDNA sensors) recognize foreign viral signatures or nucleic acids.

Regardless of the type of PRRs recognition that occurs to initiate signaling, the pathways all require the downstream activation of critical transcription factors called interferon regulatory factors (IRFs) (3). IRFs are considered the major contributors driving IFN expression, but they function in coordination with other transcription factors such as NF-κB and AP-1. There are 9 members of the IRF family, IRF1 through IRF9, which have various cell-type specific expression and functions (4). Recognition of cytosolic dsDNA and dsRNA by these cytosolic sensors activates adaptor molecules, such as STING and MAVS, respectively, which recruits and activates TBK1. Activation of TBK1 phosphorylates IRFs, which dimerize, translocate to the nucleus. IRFs that are considered positive regulators of IFN expression, such as IRF1, IRF3, and IRF7, can then bind to the interferon-stimulated response element (ISRE) in the promoter region of IFN genes to initiate transcription (3). Upon translation, type I and type III IFN proteins are secreted from the cell and bind to their respective receptors in an autocrine and paracrine manner. Receptor binding initiates a secondary signaling pathway, resulting in the expression of hundreds of antiviral interferon stimulated genes (ISGs), which inhibit viral function or confer a cellular defense against infection (1). Although the functions of type I and type III IFNs overlap, there are many distinctions between them, and regulation of type I and type III IFNs and the IFN response is often cell-type, and/or stimulus specific (1). The type I IFN response is considered more potent, inflammatory, and systemic due to ubiquitously expressed type I IFN receptors (5). In contrast, the type III IFN response is less inflammatory because their receptors are restricted to epithelial cells, and they do not upregulate certain pro-inflammatory ISGs such as IRF1 (6). Of note, there are IRF-independent mechanisms that can lead to basal or constitutive IFN production. Expression of basal IFN-β, for instance, relies more upon the AP1 component c-Jun, along with NF-κB components; similarly, c-Fos, another AP1 component, can influence production of IFN-β, in the absence of IRFs in osteoclasts (7, 8).

As our first physical line of defense to the environment, our skin is constantly exposed to pathogens. Keratinocytes are the main cellular component of the outer most layer of the skin, the epidermis, and are the target cell for many skin-infecting pathogens, including types 1 and 2 herpes simplex viruses, varicella-zoster virus, Zika virus, Dengue virus, West Nile virus, vaccinia virus, enteroviruses, and papillomaviruses (PV) (9). Therefore, keratinocytes are considered “sentinels, ” because they can recognize pathogen invasion, and are able to mount an immediate, innate IFN antiviral response to combat infection (10). Despite the importance of keratinocytes in IFN-driven innate immunity, and the fact that IFN expression and regulation has been shown to be cell-type specific, little is known about the IFN response and regulation of IFNs by IRFs within these cells. In addition, development of therapeutics for skin diseases requires characterization and knowledge of IFN regulation and function, especially considering many of these pathogens interact with, and target specific molecular components of the IFN signaling pathways to inhibit or evade IFN immunity (11–15).

Here, we aimed to compare the role of IRFs in regulating IFNs and the IFN response within keratinocytes using a canine model. Dogs are a widely used large animal model for studying human diseases. They can spontaneously develop numerous disorders homologous to their human counterpart, including cancer, genetic diseases, and immune-mediated, inflammatory, or viral skin diseases such as atopic dermatitis, systemic lupus erythematosus (SLE), dermatomyositis (DM), and papillomavirus infections (PV) (16–21). The dog is an ideal large animal model to study the host IFN-driven innate immune response to skin pathogens. The dog is immunologically more similar to the human than the laboratory mouse, which is arguably the most common model for studying disease and immunity (20). In addition, dogs have been shown to express ISGs, type I IFNs (5 subtypes of IFN-α, IFN-β, IFN-ε, IFN-κ, but not IFN-ω), and three type III IFN-λs, whereas the mouse expresses type I IFNs, but only functional type III IFN-λ2 and IFN-λ3 (22–27). This is especially relevant because type I IFNs are commonly used as a therapeutic treatment for some of these shared skin diseases, such as PV infections, and research is currently underway to investigate the therapeutic potential of type III IFNs, in both humans and dogs (25, 28, 29). The type III IFNs have the potential to have less adverse side-effects, as they are less pro-inflammatory and their receptors are primarily restricted to epithelial cells (30). IRFs have also been identified as an attractive therapeutic target due to their significant impact on IFN expression and additional roles in tumor suppression (31). Thus, we proposed to compare IRF1, IRF3 and IRF7’s impact on IFN regulation between human and canine keratinocytes, to directly address human keratinocyte-specific IRF function in IFN expression, and also to advance the dog model. IRF1, IRF3, and IRF7 were chosen because these three IRFs have been shown to be positive regulators of the IFN response within epithelial cells (32–35).

We found that, compared to canine keratinocytes, human keratinocytes are “primed” with higher basal expression of inflammatory type I and ISGs, they induce significantly higher levels of type III IFNs upon stimulation, and they express overall higher copy numbers of all investigated IFNs and ISGs compared to dog. In addition, we show that IRF3 and IRF7 may be partially responsible for differences in basal IFN expression between the species but not for induced expression. However, IRF regulation of induced IFNs, particularly IRF1, was species-specific and in some cases, stimulus-specific.

## 2 Materials and methods

### 2.1 Cell culture and ligand stimulation experiments

All canine keratinocytes, including spontaneously immortalized canine primary epidermal keratinocytes (CPEKs; Zenbio, Raleigh, NC, USA) (36) were routinely maintained in canine keratinocyte media CnT-09 (ZenBio, Raleigh, NC, USA) with 1% penicillin-streptomycin antibiotics (Sigma-Aldrich, St. Louis, MO, USA). CPEKs were utilized at passages <25, whereas the other 3 canine primary cultures, derived from normal skin of adult dogs, were used at passages <10. Normal primary human epidermal keratinocytes-neonatal (NHEKs; Lonza, Walkersville, MD, USA), normal primary human epidermal adult keratinocytes (NHEK-Adult; Lonza, Walkersville, MD, USA), and human hTERT-immortalized keratinocytes (hTERT-KerCT, which we will refer to as hTERTs; ATCC, Manassas, VA, USA) were maintained in KGM Gold Keratinocyte Growth Medium (Lonza, Walkersville, MD, USA), and utilized at passage <6. Normal primary human epidermal keratinocytes (HEKn; Lifeline Cell Technology, Frederick, MD, USA) were maintained in DermaLife basal media with Dermalife K LifeFactors supplements (Lifeline Cell Technology, Frederick, MD, USA). Normal primary dog epidermal keratinocytes were previously derived (26). In short, these primary cultures were obtained from discarded fresh biopsy samples from dogs without a history of skin disease submitted to the anatomic pathology service at the Veterinary Teaching Hospital at the University of California, Davis. Samples were collected under the approval of the UC Davis Clinical Trial Review Board for tissue banking samples and use for non-invasive procedures with a general owner consent form. Cells were isolated and maintained as previously described (26). Madin-Darby canine kidney (MDCK) cells were purchased from American Type Culture Collection (ATCC, Manassa, VA, USA) and maintained in Eagle’s minimum essential medium (ThermoFisher Scientific, Waltham, MA) containing 10% fetal bovine serum and 1% penicillin-streptomycin antibiotics (Sigma-Aldrich, St. Louis, MO, USA). HEK293 cells were purchased from American Type Culture Collection (ATCC, Manassa, VA, USA) and maintained in Dulbecco’s modified Eagle’s medium (ThermoFisher Scientific, Waltham, MA) supplemented with 10% fetal bovine serum and 1% penicillin-streptomycin antibiotics (Sigma-Aldrich, St. Louis, MO, USA. All cells were incubated at 37°C and 5% CO_2_. For ligand stimulation experiments, cells were seeded into 24 well plates at 1 x 10^5^ cells/well or 5 x 10^4^ cells per well to be subconfluent for stimulation after 24 or 48 hours respectively. To initiate antiviral interferon signaling pathways, the ligands Poly(I:C) High Molecular Weight and Poly(dA:dT) both complexed with Lyovec transfecting reagent were used (InvivoGen, San Diego, CA). Complexing to Lyovec allows ligand delivery into the cytoplasm, where it can activate cytosolic pattern recognition receptors (PRRs), as opposed to surface or endosomal compartment PRRs such as Toll-like receptors (TLRs). Poly(I:C)/lyovec, a synthetic analog of dsRNA, is therefore sensed by cytosolic RNA sensors to activate RIG-I or MDA-5 in the RIG-I-like receptor (RLR) signaling pathways. Poly(dA:dT) is a synthetic analog of dsDNA, and is sensed by cytosolic DNA sensors to activate the interferon signaling pathways. Ligands were reconstituted with endotoxin-free water as directed by the manufacturer. 24–48 hours after cell seeding, cells were mock stimulated or stimulated with 750 ng/ml of Poly(I:C) or Poly(dA:dT). Cells were then incubated at 37°C for 4–48 hours post-stimulation, as indicated for each experiment, before lysis for RNA extraction.

### 2.2 Quantification of gene expression

At the indicated times post-stimulation, cell lysates were homogenized using QIAshredders (QIAGEN Sciences Inc, Germantown, MD) and total RNA was isolated using the RNeasy Mini Kit (QIAGEN). Complementary DNA (cDNA) was synthesized, and contaminating DNA was removed using the QuantiTect Reverse Transcription Kit (QIAGEN). cDNA was diluted with nuclease-free water to a stock concentration of 5 ng/μl for quantitative real time PCR. Real time PCR using QuantiTect SYBR Green detection (QIAGEN) was performed for the following genes for both human and canine: Ribosomal Protein L13a (RPL13A), IFN-β, IFN-λ1, IFN-λ2, IFIT1, DAI (also referred to as ZBP1), IRF7, IRF1, and IRF3. Primer sequences and efficiencies are listed in **Table 1**. Exact designated nomenclature for canine IFN-λ1 and IFN-λ2 in GenBank remains unclear, and therefore these genes will correspond to GenBank accession numbers NM_001114853 and XM_014117025.2 respectively, as described previously (37). All primers were custom designed using Primer3 software (Whitehead Institute, Cambridge, MA, USA). A single PCR reaction mixture contained 12.5μl Sybr green master mix, 0.2μM final concentration of 10μM forward (F) and reverse (R) primer (0.5μl each), 10ul of 5ng/μl cDNA, and brought up to a total reaction volume of 25μl with nuclease-free water. Amplification and gene expression detection was performed in 96 well plates using the Roche LightCycler 480 system under the following conditions: 95°C for 13.5 minutes, then 50 cycles of 95°C for 10 sec, 57°C for 60 sec, followed by a melting curve protocol of 5 sec at 95 °C, 60 sec at 50 °C, heating to 97 °C, and cooling for 30 sec at 37 °C. Relative fold mRNA expression was calculated using the ΔΔCt method (38), relative to the unstimulated or non-targeting control, which was set to 1, and normalized to the expression of the reference gene, RPL13A.

**Table 1:** Primer sets and efficiencies for reverse transcriptase real time PCR (RT-qPCR).

Canine target gene	Primer sequence forward and reverse (5' to 3')	Efficiency (%)	Size (bp)
RPL13A (reference gene)	TGGGCCGGAAGGTTGTAGTCGT	97	94
	TTGCGGAGGAAGGCCAGGTAATTCA		
IFN-β	AAGCAGCAGCAGTTTGGAGT	98	118
	CTGGCGTGATTTCTCGATTT		
IFN-λ1	TCCCTACTTCCAAACCCACC	100	131
	GTTCTTCCAGGAGAGCGACT		
IFN-λ2	CGCCTCTTCCCTAGAAACCGGGACC	100	99
	CTCCAGGACCTTCAGTGTCAAGGCC		
IFIT1	TGGCAGTGCAGAGGTCAGGATAG	94	119
	GCCAGGTGTATATAAGCAATGTCAA		
DAI	TCACTGACCAGCATTCGAGAG	98	99
	TGTGGTTATGTGGTCCGTT		
IRF1	GAGCCAACATGCCCATCAC	93	100
	TCCTCTTTATTAATCCAGATCAGCC		
IRF3	GATGGAGACCTGTTCGACCTG	100	83
	GTGTAGCGTGGTGAGTGTC		
IRF7	GCAAGGTCTACTGGGAGGTG	91	106
	GTGCTGAAGTCGAAGATGGG		
Human target gene	Primer sequence forward and reverse (5' to 3')	Efficiency (%)	Size (bp)
RPL13A (reference gene)	GGGGCATCTGTTGGACTTTC	98	141
	CCTTGGTTGTGCATCTTCCC		
IFN-β	CAGGTAGTAGGCGACACTGT	100	79
	AGAAGCACAACAGGAGAGCA		
IFN-λ1	CATCCTCTCCCAGCTCCAG	93	149
	GGTTGAAGGTGACAGATGCC		
IFN-λ2	TAGACATGACTGGGGACTGC	99	138
	GGGACTTGAACTGGGCTATG		
IFIT1	GCTTGAAGTGGACCCTGAAA	100	116
	GGGGAAGCAAAGAAAATGGC		
DAI	CCTCTCAGGGCCACAGTG	95	62
	AGCTGCAAGGAGTCGGAG		
IRF1	CACTCCTTAGTCGAGGCAAG	97	128
	GAAGAGGGAAGAAGGCAGAG		
IRF3	CGGCTTTTGGGTCTGTTAC	94	139
	CTCCGGGTAGCTCTCAAACT		
IRF7	CTCCCCACGCTATACCATCT	100	92
	GTTCCAGCTTCACCAGGAC		

For absolute quantitative PCR to determine gene copy number, standard curves for each gene were generated using six 10-fold dilutions of known DNA concentrations. Template DNA for each gene’s standard curve was generated by conventional PCR amplification of CPEK or NHEK cDNA using the primers listed in **Table 2**. The PCR product was then purified by gel extraction using the QIAquick gel extraction kit (Qiagen), sequenced to ensure correct template amplification, and quantified using a nanodrop spectrophotometer (Thermo Scientific). Copy numbers of each standard curve template were calculated and used as the starting point for the 10-fold dilutions. Copy number of basal and induced gene expression was normalized to 10^4^ copies of the reference gene RPL13A.

**Table 2:** Primer sets for Conventional PCR.

Canine target gene	Primer sequence forward and reverse (5’ to 3’)	Size (bp)
RPL13A (reference gene)	CCTGTTTCAAGGGATAAGA	314
	ACTGTCCGCCAAAAGATACG	
IFN-β	TGCATCCTCCAAACAACTCTC	481
	TTGTCCAGGCACAGATGCTG	
IFN-λ1	CTACAGCTCGGGTCCTGGT	516
	CGGAAGAGGTTGAACGTGAT	
IFN-λ2	CAGGTTCAAATCTCTGTCACC	267
	AGCTGGGAGTGGATGTGGC	
IFIT1	CTCCTAAAAATGGCCCAACA	560
	TACTCCAGGGCTTTGTTCAA	
DAI	GAGGTGCCAGCAAGACAT	149
	TGTGGTTATGTGGTCCGTT	
IRF1	TGCGGAGCCAACATGCCCAT	310
	TCCTGCTCTGGTCCTTCACT	
IRF3	CTCGGATACCCAGGAAGACA	714
	GTGGGAACAACCTTGAGCAT	
IRF7	GACGCCCTCATCTTCAAGG	1321
	GGTCCTCGTAGAGGCTGTTG	
Human target gene	Primer sequence forward and reverse (5’ to 3’)	Size (bp)
RPL13A (reference gene)	ACATAGGAAGCTGGGAGCAA	350
	AAATACAGGTGAGGAGCATG	
IFN-β	TCGAAGCCTTTGCTCTGGCA	529
	TCCTTGGCCTTCAGGTAATG	
IFN-λ1	GACTTTGGTGCTAGGCTTGG	588
	TAAGGTGTGGGGTGTCAGGT	
IFN-λ2	GACAAGACCCAAACAGACC	796
	TTTTCCTGGAGGTGAGTTGG	
IFIT1	ACACCTGAAAGGCCAGAATG	703
	CTTGTAGCAAAGCCCTATCT	
DAI	CTGCAAGAAGCACCAGGCTC	523
	ACCAAGGCACAGAGAGAGGA	
IRF1	CGCCACTCCTTAGTCGAG	211
	TGCAGGAGCGATTCGGC	
IRF3	CGGTCTCCTCCACTGAACTC	600
	GACGCCTCCTCTTTTCCTCT	
IRF7	CAGATCCAGTCCCAACCAAG	801
	GCTCCATAAGGAAGCACTCG	

### 2.3 CRISPR-Cas9 knock out

hTERTs and CPEKs were seeded into 6-well plates at 2 x 10^5^ cells per well in preparation for CRISPR IRF1 and IRF3 lentiviral transduction at 50-80% confluency. Edit-R All-in-one lentiviral sgRNAs, which combines gene-specific, targeted guide RNA (sgRNA) and Cas9 nuclease expression into a single lentiviral vector were purchased from Horizon Discovery Ltd (Cambridge, UK). Lentiviral sgRNA non-targeting control #1 (NTC) was also purchased as a scrambled control. Human sgRNA target sequences were as follows: IRF1, GCATGGCTGGGACATCAACA; IRF3, GGTTGTGCCCACGTGCCTCA. Canine sgRNA target sequences were as follows: IRF1, GCATGGCTGGGACATCAACA; IRF3, GTGTACTGAGCTGCTTGGGT. Human CRISPR sgRNA IRF 1 and IRF3 lentiviral particles were transduced into hTERTs at 0.8 multiplicity of infection (MOI) according to the manufacturer’s instructions. Canine CRISPR sgRNA IRF1 and IRF3 lentiviral particles were transduced into CPEKs at 5 MOI according to the manufacturer’s instructions. NTC lentiviral control sgRNA was also transduced into both hTERTs and CPEKs at the same MOI as targets. For both cell types, cells were incubated at 37°C for 24 hours, at which time, transduction medium was removed and replaced with selection medium containing 1μg/ml puromycin. Selection media was changed every other day for 3–4 days, until non-transduced control cells died. Successfully transduced cells were expanded in growth media for subsequent experiments, and gene editing and knock out was verified using a genomic cleavage mismatch assay, and western blot for IRF1 and IRF3 protein knock out (KO).

### 2.4 shRNA transduction

For transduction into hTERTs and HEK293s, MISSION Lentiviral transduction particles specific for human IRF7 were purchased (validated target sequence GCTGGACGTGACCATCATGTA), along with MISSION shRNA control transduction particles (shRNA NTC) (Sigma-Aldrich). Human shRNA IRF7 lentiviral particles were transduced into hTERTs at 1.6 MOI and HEK293s at 3.0 MOI according to manufacturer’s instructions. Lentiviral control shRNA particles were also transduced at the same MOI. Cells were incubated for 24 hours, then transduction media was replaced with fresh growth media for another 24 hours before the addition of selection media containing 1μg/ml puromycin. Selection media was changed every other day for 3–4 days, until non-transduced control cells died. Successfully transduced cells were expanded in growth media for subsequent qRT-PCR experiments. IRF7 KD was verified by measuring IRF7 mRNA expression. For CPEKs and MDCKs, MISSION Lentiviral transduction particles specific for mouse IRF7 were purchased, as there are no commercially available IRF7 shRNA lentiviral particles specific for dog, and the mouse IRF7 target sequence (CTTCGACTTCAGCACTTTCTT) is 95% homologous to dog IRF7. shRNA transduction into CPEKs and MDCKs was performed as described above, except particles were transduced at 5 MOI. Successfully transduced cells were expanded in growth media for subsequent qRT-PCR stimulation experiments. IRF7 KD was verified by measuring IRF7 mRNA expression.

### 2.5 Western blot

Upon completion of CRISPR IRF1 and IRF3 KO experiments, NTC, IRF1 KO, and IRF3 KO hTERT or CPEK whole cell lysates were collected by scraping cells from 6-well plates, after a 5 minute incubation on ice with RIPA buffer (Sigma, Aldrich) containing protease and phosphatase inhibitors (Thermo Fisher Scientific). To detect IRF1 in western blots, sub-confluent wells of NTC control and IRF1 KO cells were stimulated for 4–6 hours with either PBS with 0.1% BSA, or 20ng/ml human TNF-α (PeproTech, NJ, USA) prior to collection in RIPA buffer as above. Cells were then centrifuged at 10, 000 rpm for 10 min, and clarified supernatants were transferred to a separate tube for storage at -80°C until use. Protein concentration was determined using the Pierce bicinchoninic acid (BCA) protein assay kit (Thermo Scientific). Equal amounts of protein for each sample were combined with 2X Laemmli sample buffer (BioRad Laboratories, Hercules, CA), then denatured at 100°C for 5 minutes before resolving on an SDS-PAGE gel. Proteins were then electrotransferred onto nitrocellulose membranes, blocked for 1 hour with 5% milk blocking buffer, and probed with primary antibodies against IRF1 (Cat #8478, CST, Danvers, MA) at 1:1000 dilution, and IRF3 (Cat #11312-1-AP, Proteintech, Rosemont, IL) at 1:2000, overnight at 4°C. Membranes were washed and then probed with a HRP-conjugated rabbit secondary antibody (Life Technology, Carlsbad, CA) at 1:2500 dilution for 1 hour before detection with chemiluminescence (ECL reagent, GE healthcare) and exposure with film. To ensure equal protein load, membranes were subsequently probed with HRP-conjugated β-actin (Abcam) at a 1:40, 000 dilution. Densitometry analysis of the bands was performed using ImageJ software (NIH) and values were normalized to β-actin loading control.

### 2.6 Mismatch assay

To validate the occurrence of gene editing, and sufficient gene silencing of IRF1 and IRF3, while ensuring the absence of cross-IRF KO, verification was accomplished using a mismatch genomic cleavage assay, as well as western blot to observe reduction of IRF protein. The mismatch genomic cleavage assay detects and cleaves the CRISPR edited region of the DNA. Mismatch assay was performed on IRF1 and IRF3 CRISPR silenced, or NTC hTERTs and CPEKs using the GeneArt™ Genomic Cleavage Detection Kit (Thermofisher) following their recommended protocols using the following primers: HumanIRF1-For: AGAGCTCGCCACTCCTTAGTC; HumanIRF1-Rev: CCTTTTCCCCTGCTTTGTATC; HumanIRF3-For: GGGTGTTTTCCCTGACTCCT; HumanIRF3-Rev: CATGCATTGAGCACCTGAGT; DogIRF1-For: GCTCCTGCTAAAACCCTGTG; DogIRF1-Rev: ATCCAGGCTCACAACTGCTT; DogIRF3-For: ACAAGACCGTGATGGGCTAC; and DogIRF3-Rev: AACCTTAAGCAGGCTCCACA. Gene edited cells were routinely passaged and seeded into 6-well plates at 1 X 10^5^ cells/ml in 1.5 mls cell culture media (KGM for human cells and CnT-09 for canine cells). When cells reached confluency, the wells were washed with PBS and cell pellets collected into 1.5ml tubes and stored at -80°C until use. Cells were lysed according to manufacturer’s recommended protocols, where 50μl Cell Lysis Buffer containing 2μl Protein Degrader was mixed with each cell pellet, placed into a thermocycler, and run with the following program: 68°C for 15 min; 95°C for 10 min; 4°C hold. PCR was then performed using 2μl cell lysate, 1μl of 10μM dilution of primers, 25μl AmpliTaq Gold 360 master mix, in a total volume of 50μl. Amplification was performed using the Veriti 96-well Thermal Cycler (Applied Biosystems) under the following conditions: 95°C for 10 minutes, then 50 cycles of 95°C for 30 sec, 57°C for 30 sec, and 72°C for 30s, followed by a final extension at 72 °C for 7 minutes. The PCR product (3μl) was combined with 1μl Detection Reaction Buffer and 5μl water and run on a thermal cycler with the following program: 95°C for 5 min, followed by a decrease in temperature from 95°C to 85°C at -2°C/sec and then a decrease in temperature from 85°C to 25°C at -0.1°C/sec. Detection Enzyme (1μl) was then added to all samples and incubated at 37°C for 1 hour. The entire reaction volume was then loaded onto a 3% agarose gel containing SYBRsafe DNA gel stain (Thermofisher) for 30 minutes at 70V and visualized using a UV transilluminator (Biorad ChemiDoc MP Imaging System).

### 2.7 Statistical analysis

Statistical analysis and graphical representation of data was performed using GraphPad Prism 9 (GraphPad software, San Diego, CA, USA). To calculate statistical differences between the means of different groups, student unpaired t-tests or one way analysis of variance (ANOVA) were used as appropriate. Values of *P* < 0.05 were considered statistically significant.

## 3 Results

### 3.1 Induction of type I and type III IFNs and ISGs in keratinocytes differs between human and dog

**Figure 1:**
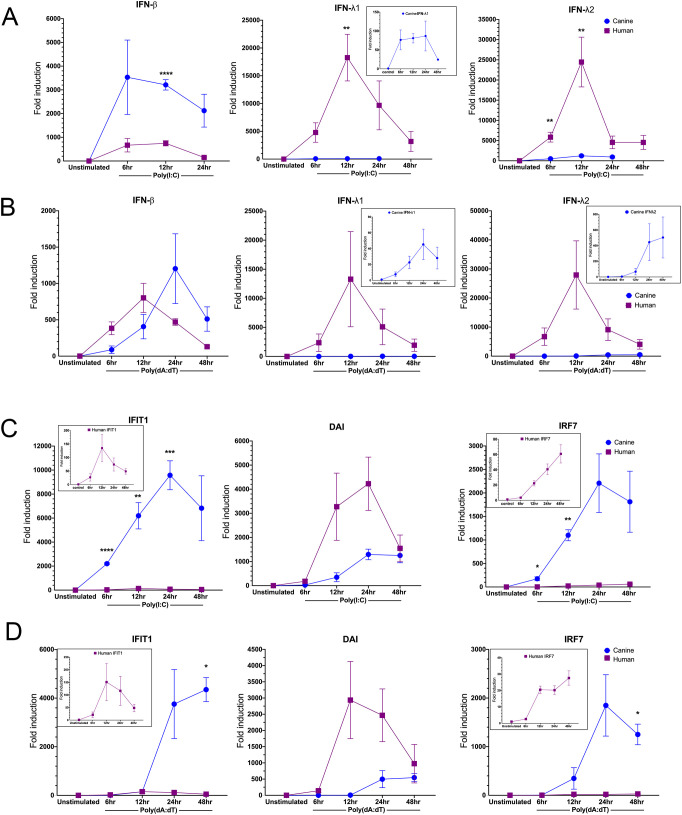
Induction of type I and type III IFNs and ISGs in human and canine keratinocytes after stimulation with Poly(I:C) and Poly(dA:dT). Human (NHEK) and canine (CPEK) keratinocytes were stimulated with analogs of dsRNA [Poly(I:C)lyovec] and dsDNA [Poly(dA:dT)] to initiate the antiviral interferon signaling pathways. Quantitative RT-PCR was used to assess mRNA expression of IFNs and ISGs at different timepoints between 6–48 hours after stimulation. Data is shown as fold induction (ΔΔCt) of mRNA expression over unstimulated control (set to 1), and normalized to the reference gene, RPL13A. Fold induction of type I IFN-β and type III IFN-λ1 and IFN-λ2 within NHEKs and CPEKs are shown after stimulation with Poly(I:C) **(A)** or Poly(dA:dT) **(B)**. Fold induction of the ISGs IFIT1, DAI, and IRF7 within NHEKs and CPEKs are shown after stimulation with Poly(I:C) **(C)** or Poly(dA:dT) **(D)**. Each data point represents the mean value from 3 [Poly(I:C) data] or 5 [Poly(dA:dT) data] independent experiments, performed in duplicate, ± standard errors of the mean (SEM). Asterisks (*) indicate significance (*p* < 0.05) between human and canine fold induction at each time point, where *p < 0.05; **p < 0.01; ***p < 0.001; ****p < 0.0001 using multiple unpaired t-tests.

To determine the optimal dose of ligand to maximize gene expression and limit toxicity to cells a dose response curve was performed on both normal neonatal human epidermal keratinocytes (NHEKs) and canine primary epidermal keratinocytes (CPEKs) (**Supplementary Figure 1**). We found that expression of Interferon Induced Protein With Tetratricopeptide Repeats 1 (IFIT1), an ISG that is significantly upregulated in CPEKs after stimulation, in NHEKs resulted in minimal IFIT1 induction. We alternatively measured induction of type III IFNs in NHEKs and observed a robust induction after stimulation with both Poly(I:C) and Poly(dA:dT). For both dog and human the dose response curve plateaued at approximately 1000ng/ml, and we chose 750ng/ml as the optimal dose. The drastic difference in IFIT1 expression between NHEKs and CPEKs led us to pursue the hypothesis that induction of IFN and/or ISG expression may not only be cell-type specific but also species specific, which has important implications when deciding on an appropriate animal model to study interactions between human infectious diseases and innate immunity. To this aim, we performed a stimulation time course to compare the kinetics of the interferon response between dog and human keratinocytes. We chose to examine induction of type I and type III IFNs as well as three ISGs, including IFIT1, DAI and IRF7. After Poly(I:C) stimulation, CPEKs had a significantly robust induction of type I compared to NHEKs and also compared to type III (**Figure 1A**). In contrast, NHEKs had lower induction of type I IFN but high induction of type III IFNs compared to CPEKs (**Figure 1A**). Upregulation of type I IFN and type III IFNs occurred concurrently in both dog and human keratinocytes. In NHEKs, expression of both types of IFNs peaked consistently at 12 hours post stimulation, whereas peak upregulation of IFNs in CPEKs was more variable, but averaged around 24 hours post stimulation (**Figure 1A**). When stimulated with Poly(dA:dT), IFN expression results were very similar to Poly(I:C) results, with the exception that peak induction was lower for both CPEK and NHEK, apart from IFN-λ2 in NHEKs (**Figure 1B**). Differences were also seen in ISG upregulation after stimulation. In CPEKs after Poly(I:C) treatment, there was robust upregulation of all three examined ISGs, peaking approximately 12–24 hours after peak IFN induction, with significantly higher upregulation of IFIT1 and IRF7 compared to NHEKs (**Figure 1C**). Human NHEKs had a different ISG response, with robust upregulation of DAI compared to CPEKs, and only minimal upregulation of IFIT1 and IRF7. Peak upregulation of ISGs in NHEKs varied from 12–24 hours with IRF7 remaining low 48 hours post stimulation (**Figure 1C**). Results after stimulation with Poly(dA:dT) were similar to Poly(I:C) results with the exception that peak fold induction tended to be lower (**Figure 1D**). To ensure the differences between IFN and ISG induction between human and dog were not due to the different optimized culture media between NHEKs and CPEKs, a culture of CPEKs was gradually adapted to the NHEK KGM cell culture media and utilized for a ligand stimulation experiment. Gene expression of the IFNs and ISGs maintained the same patterns seen in CPEKs cultured in their own growth media (data not shown), supporting the conclusion that human keratinocytes express higher induced type III IFNs compared to dog, and that dog keratinocytes express higher induced type I IFNs compared to humans.

### 3.2 Differences in induced IFN and ISG expression between stimulated human and canine cells is consistent across keratinocytes from multiple sources

**Figure 2:**
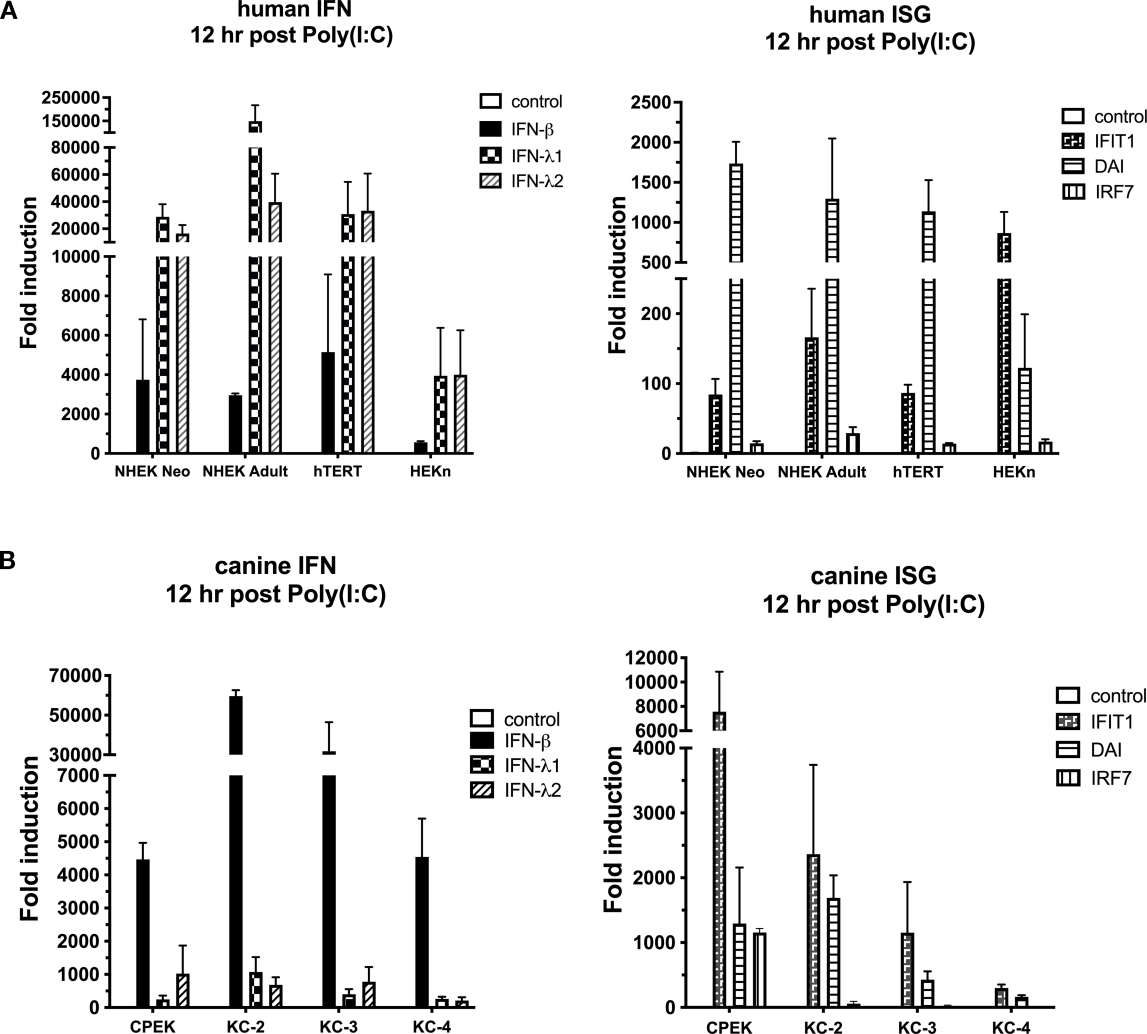
Induction of type I and III IFN and ISGs in human and canine keratinocytes from multiple sources. **(A)** Human cells lines NHEK (normal pooled neonatal foreskin primary keratinocytes, Lonza), NHEK Adult (normal primary keratinocytes derived from a cutaneous site on an adult donor), hTERT/Ker-CT (hTERT-immortalized keratinocytes), and HEKn (normal human neonatal foreskin primary keratinocytes, Lifeline Cell Technology) were stimulated with Poly(I:C), and mRNA expression of IFNs and ISGs was assessed 12 hours after stimulation. **(B)** Canine cell lines CPEK (Zenbio, Raleigh, NC), and three primary canine keratinocyte cell lines derived from the skin of normal dogs, KC-2, KC-3, and KC-4, were also stimulated with Poly(I:C) and mRNA expression of IFNs and ISGs were assessed 12 hours after stimulation. Data is shown as fold induction (ΔΔCt) of mRNA expression over unstimulated control (set to 1), and normalized to the reference gene, RPL13A. Results are expressed as the mean of 3 independent experiments, performed in duplicate, ± SEM.

Next, we asked the question, is this difference in IFN and ISG expression after Poly(I:C) or Poly(dA:dT) stimulation shared among other keratinocytes of the same species. Further, for human keratinocytes, we wanted to know if this pattern of expression was due to age (neonatal versus adult) or site differences (dry skin vs foreskin-derived), and if an immortalized keratinocyte cell line which is widely used to study PV infections also exhibits the same pattern. Only results from Poly(I:C) 12 hours post stimulation are shown. As expected, the magnitude of fold induction is variable between experiments, however, all four human cultures consistently exhibited the same IFN expression pattern, with between 5 and 50 times higher induction of type III IFNs compared to type I IFN (**Figure 2A**). The induction of ISGs was also similar between cell types, with minimal induction of IRF7, moderate induction of IFIT1 and robust induction of DAI, with the exception of HEKn cells, whose pattern for IFIT1 and DAI was slightly different (**Figure 2A**). For dog, induction of IFNs was also similar between dog keratinocyte sources: between 4 and 55 times higher upregulation of type I IFN compared to type III IFNs. For the ISGs, results were more variable but consistently showed higher expression of IFIT1 compared to DAI (**Figure 2B**). Results were similar for Poly(I:C) 24 hours post stimulation, as well as Poly(dA:dT) at 12 and 24 hours post stimulation (data not shown).

### 3.3 Basal expression of type I and type III IFNs and ISGs differs between human and canine keratinocytes

**Figure 3:**
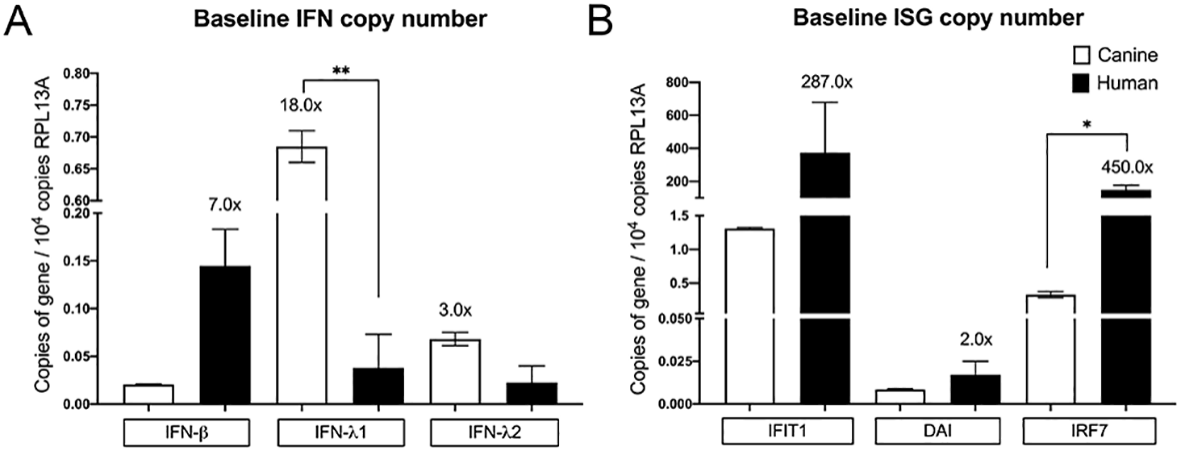
Basal expression of IFNs and ISGs in human and canine keratinocytes. Absolute quantitative real-time PCR for mRNA expression of type I IFN-β, and type III IFN-λ1, IFN-λ2 **(A)**, as well as ISGs IFIT1, DAI, and IRF7 **(B)** was performed on RNA extracted from unstimulated NHEKs and CPEKs lysed 24 hours after seeding. Data is shown as gene copy number normalized to 10^4^ copies of the reference gene RPL13A, and represents the mean of 2 independent experiments, performed in duplicate, ± SEM. Asterisks (*) indicate a significant difference (*p* < 0.05) in expression between NHEKs and CPEKs, where *p < 0.05; **p < 0.01 using multiple unpaired t-tests.

Because induced expression of IFNs and ISGs differed between NHEKs and CPEKs, we then inquired whether the same holds true for basal expression of IFNs and ISGs. Interestingly, the dog had significantly higher basal copies of type III IFNs compared to human, and compared to type I IFN, whereas in contrast, human had higher basal type I IFN compared to dog and compared to type III IFNs (**Figure 3A**). In addition, human NHEKs had higher basal copies of all the ISGs compared to dog, but particularly IFIT1 and IRF7, whereas dog CPEKs had low expression of all ISGs, especially when compared to human (**Figure 3B**).

### 3.4 Basal IFN and ISG expression affects relative fold induction of expression, but not total induced copy number

**Figure 4:**
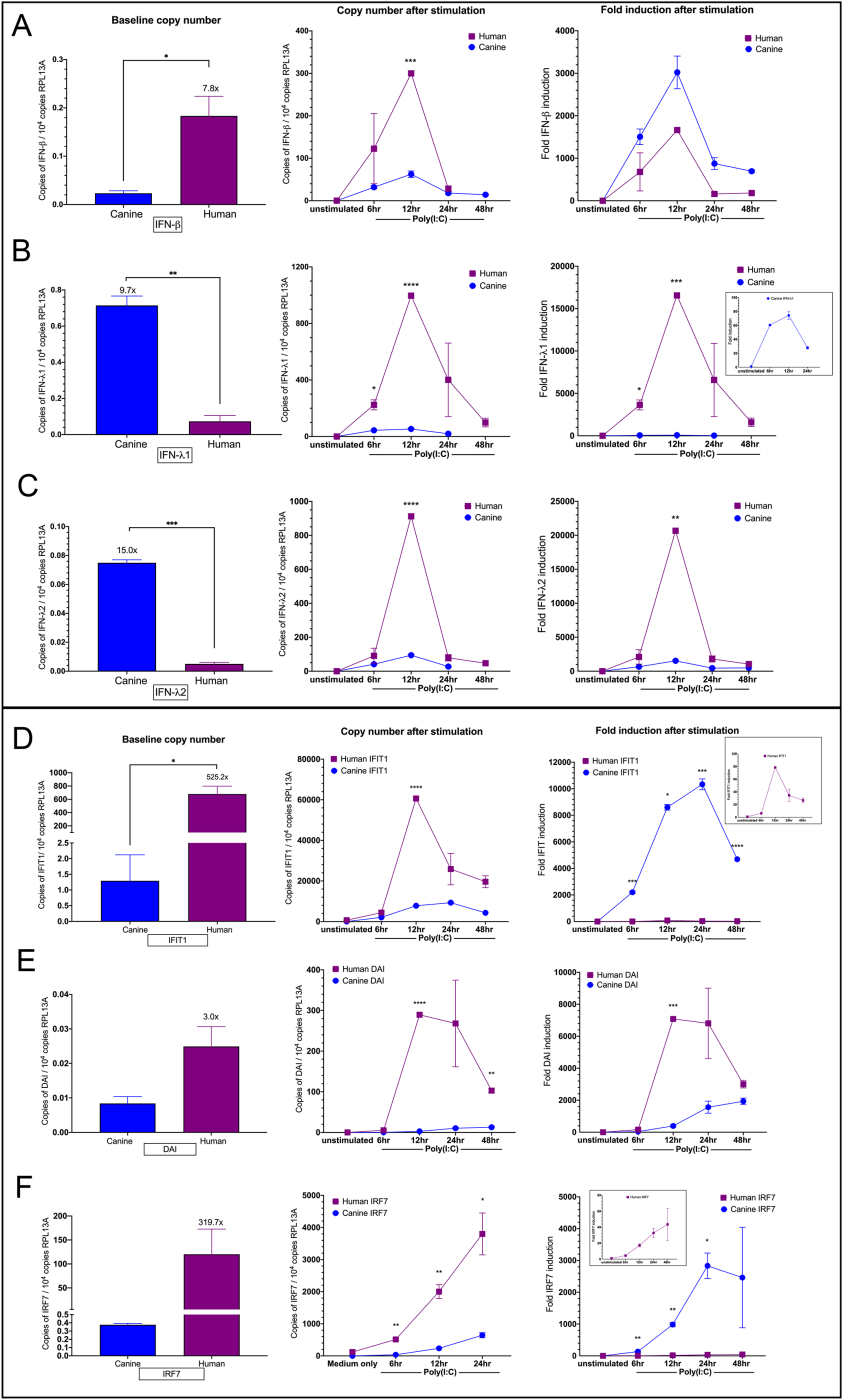
Absolute copy number and fold expression of IFNs and ISGs in human and canine keratinocytes at baseline and after Poly(I:C) stimulation. Basal copy number, induced copy number after Poly(I:C) stimulation, and relative fold induction after Poly(I:C) stimulation of IFN-β **(A)**, IFN-λ1 **(B)**, IFN-λ2 **(C)**, IFIT1 **(D)**, DAI **(E)** and IRF7 **(F)** in human NHEKs and canine CPEKs are shown. Gene copy number was normalized to 10^4^ copies of the reference gene RPL13A. Relative fold induction is shown as ΔΔCt of mRNA expression over unstimulated control (set to 1), and normalized to RPL13A. Data is presented as mean ± SD of one representative experiment of two. Asterisks (*) indicate a significant difference (*p* < 0.05) in expression between NHEKs and CPEKs, where *p < 0.05; **p < 0.01; ***p < 0.001; ****p < 0.0001.

Given the differences in basal and induced IFN and ISG expression between dog and human keratinocytes, we wanted to determine if the basal expression influenced the fold induction and final induced copy number. To this aim, we performed concurrent absolute copy number and fold change analysis using the data from a single representative experiment utilized in the combined results of 2 to 5 experiments shown in **Figures 1**, **3**. As such, absolute basal data presented here is similar to **Figure 3**, and fold induction data is similar to **Figure 1**, but slight numerical differences are a result of the analysis of a single experiment rather than the mean of 2 to 5 experiments. Thus, results from a single data set were analyzed comprehensively, to determine basal copy number and total copies after induction, as well as relative fold change after induction. Basal IFN-β copy number was 7.8x higher in NHEKs compared to CPEKs; however, CPEKs had higher fold induction of type I IFN after Poly(I:C) stimulation (**Figure 4A**, column 1 and column 3). In contrast, for IFN-λ1 and IFN-λ2, CPEKs had 9.7x or 15x higher basal copy number respectively, but induced fold expression of NHEK IFN-λ1 and IFN-λ2 was significantly higher after Poly(I:C) stimulation (**Figures 4B, C**, column 1 and 3). This pattern of low basal expression resulting in high fold induction was also seen with the ISGs. NHEKs had significantly higher (525.2x) basal IFIT1 copies than CPEKs, yet after Poly(I:C) stimulation, NHEKs were only upregulated up to 80x higher compared to unstimulated cells, whereas CPEKs were upregulated over 10, 000x compared to unstimulated cells, a significant difference at each time point compared to NHEKs (**Figure 4D**, column 1 and 3). NHEKs also had dramatically higher (319.7x) basal IRF7 copies than CPEKs, yet after stimulation, NHEKs were only upregulated up to approximately 45x higher than unstimulated cells and CPEKs were upregulated close to 3000x higher than unstimulated cells, significantly higher than NHEKs (**Figure 4F**, column 1 and 3). As for DAI, basal copy numbers were very low in both NHEKs and CPEKs, with NHEKs having only 3x higher copy number. After stimulation, DAI in NHEKs were induced up to 7000x higher than unstimulated cells and DAI in CPEKs were induced up to about 2000x higher (**Figure 4E**, column 1 and 3). This data shows that dramatically different basal levels of gene expression between NHEKs and CPEKs resulted in large differences in fold induction and suggests that relative fold induction of IFNs and ISGs in keratinocytes is at least, in part, determined by basal IFN and ISG levels. NHEKs had high basal type I IFN, IFIT1 and IRF7 levels compared to CPEKs and therefore, did not require substantial induction of those genes, in contrast to CPEKs. Conversely, CPEKs had higher basal type III IFNs, thereby requiring less induction, whereas NHEKs required much greater induction. As for final copy number of IFNs or ISGs after stimulation, NHEKs had consistently, and significantly higher total copies of each gene compared to CPEKs, ranging from 5 to 30 times higher, regardless of basal expression levels (**Figures 4A–F**, column 2). These results all suggest that there may be a threshold limit to the level of induced IFN or ISG expression, and that high basal expression provides a “head start” to reaching this threshold, and results in less responsiveness to stimulation, whereas genes with low basal expression must respond more substantially after stimulation to reach the threshold. In addition, results suggest that human keratinocytes have a much higher IFN and ISG expression limit than canine keratinocytes. NHEKs and CPEKs were also induced using Poly(dA:dT) and analyzed in the same manner, exhibiting similar patterns (**Supplementary Figure 2**).

### 3.5 Canine keratinocytes have higher basal IRF3 whereas human keratinocytes have higher basal IRF7

**Figure 5:**
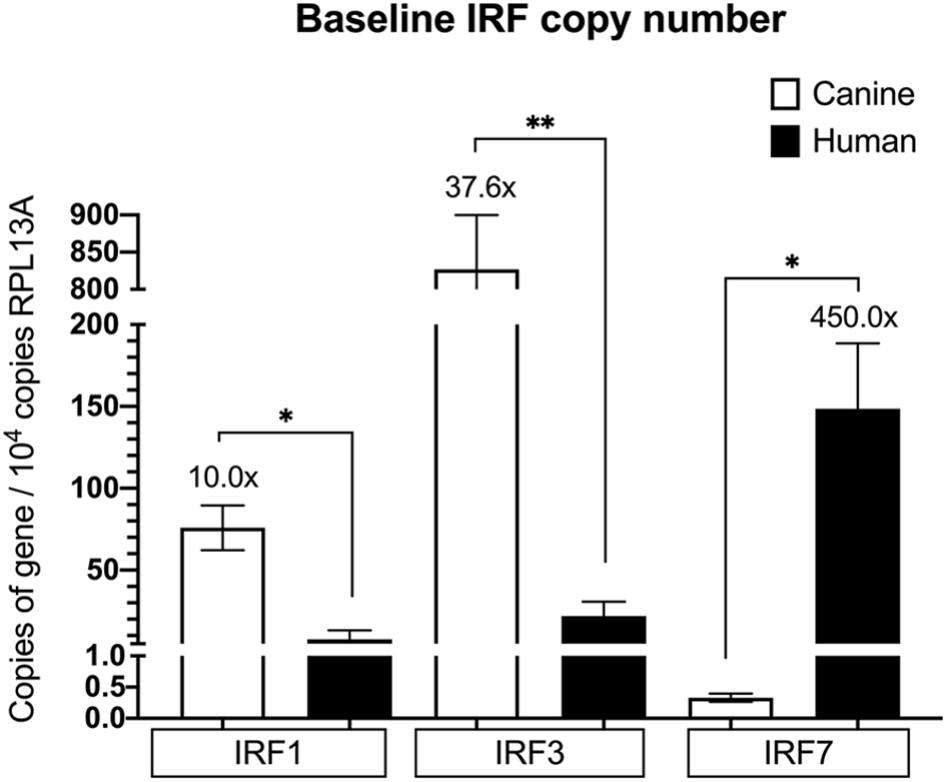
Basal expression of IRF1, IRF3 and IRF7 in human and canine keratinocytes. Absolute quantitative real-time PCR for mRNA expression of IRF1, IRF3, and IRF7 was performed on RNA extracted from unstimulated NHEKs and CPEKs lysed 24 hours after seeding. Data is shown as gene copy number normalized to 10^4^ copies of RPL13A, and represents the mean of 2 independent experiments, performed in duplicate, ± SEM. Asterisks (*) indicate a significant difference (*p* < 0.05) in expression between NHEKs and CPEKs, where *p < 0.05; **p < 0.01 using multiple unpaired t-tests.

Because IRFs are major players within the IFN signaling pathways, next we wanted to determine if basal expression of the IRFs differed between human and dog. We chose to look at IRF1, IRF3, and IRF7, as these IRFs have been commonly shown to play a major role in positive regulation of IFN expression within epithelial cells specifically (28, 30, 31, 33). Interestingly, canine keratinocytes had higher basal IRF3 (almost 40x more copies) compared to human, and lower IRF7, whereas human keratinocytes had 450x higher IRF7 compared to canine keratinocytes. In addition, the dog had 10x more copies of basal IRF1 than human (**Figure 5**). We postulated that these differing basal IRF levels between human and canine could influence the differential regulation of type I and type III IFNs in human versus canine keratinocytes and thus sought to generate canine and human keratinocytes lacking IRF expression.

### 3.6 Generation of IRF1 and IRF3 knockout human and canine keratinocytes using CRISPR gene editing and IRF7 knockdown human and canine keratinocytes using shRNAs

**Figure 6:**
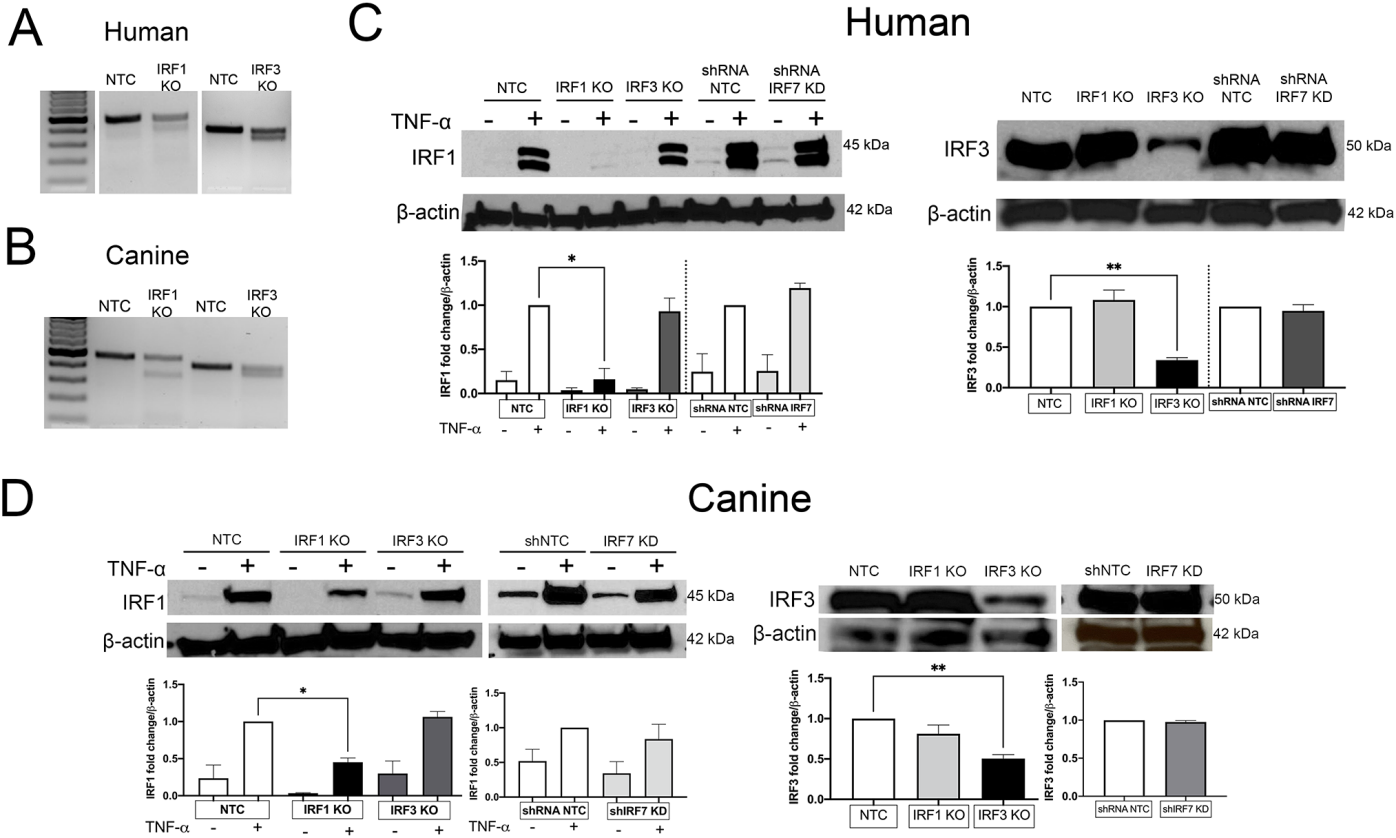
Validation of CRISPR-Cas9 knockout of IRF1 and IRF3 in human and canine keratinocytes. **(A)** Mismatch genomic cleavage detection assay indicating cleavage in IRF1 and IRF3 Knockout (KO) hTERTs and **(B)** CPEKs cells compared to NTC (non-targeting control) cells. **(C)** Western blot of control NTC cells, IRF1 KO, IRF3 KO, and shRNA-mediated IRF7 knockdown (KD) hTERTs or **(D)** CPEKs analyzed with anti-IRF1 or anti-IRF3 antibodies. To detect IRF1 protein, cells were stimulated with 20ng/ml human TNF-α for 4 (CPEKs) or 6 (hTERTs) hours. The densitometry values are presented as fold change of protein bands normalized to the bands of the β-actin loading control. Values are representative of two independent experiments and error bars indicate ± SEM. Asterisks (*) indicate a significant difference (*p* < 0.05) in expression, where *p < 0.05; **p < 0.01.

To determine the impact of IRF1 and IRF3 on IFN expression, human and canine keratinocytes with knockout (KO) of IRF1 and IRF3 were generated using CRISPR-based gene editing. hTERT immortalized human keratinocytes (hTERTs) were used in place of NHEKs because primary cells are too sensitive to survive significant changes in growth conditions and cannot be maintained long term following transduction and selection. However, as shown in **Figure 2**, hTERTs and NHEKs have similar IFN upregulation patterns, as well as basal IRF expression levels (data not shown). The mismatch genomic cleavage assay revealed gene editing occurred in the designated target region of IRF1 and IRF3 in both hTERTs (**Figure 6A**) and CPEKs (**Figure 6B**) compared to wild-type NTC cells, as depicted by a fainter full length band of unedited DNA, and the presence of smaller cleavage products. Next, IRF1 protein levels in NTC and IRF1 KO hTERTs and CPEKs were measured by western blot after stimulation with TNF-α to reach detectable amounts. IRF1 protein expression in hTERTs with KO of IRF1 was reduced by approximately 85%. IRF1 expression was not reduced in hTERTs with KO of IRF3 (**Figure 6C**). IRF1 protein expression in CPEKs with KO of IRF1 was reduced by approximately 55%. IRF1 expression was not reduced in CPEKs with KO of IRF3 (**Figure 6D**). IRF3 protein expression was also reduced by about 65% in hTERTs with KO of IRF3 and by about 50% in CPEKs with KO of IRF3. There was no significant reduction of IRF3 in CPEKs and hTERTs with KO of IRF1 (**Figures 6C, D** respectively). Taken together, we show that IRF1 and IRF3 CRISPR knockout cell lines successfully reduced protein expression, with little to no off-target IRF effects.

Due to a lack of appropriate IRF7 antibodies for western blot validation of CRISPR gene silencing, knock down (KD) of IRF7 was accomplished by performing long-term, stable shRNA transfection in both CPEKs and hTERTs. No significant non-specific knock down of IRF1 or IRF3 in the IRF7 KD cells was seen (**Figures 6C, D**). mRNA for IRF7 was determined in each experiment to verify sufficient knockdown.

### 3.7 IRF3 and IRF7 impact basal type III IFN expression in human keratinocytes and IRF3 impacts type I IFN expression in canine keratinocytes

**Figure 7:**
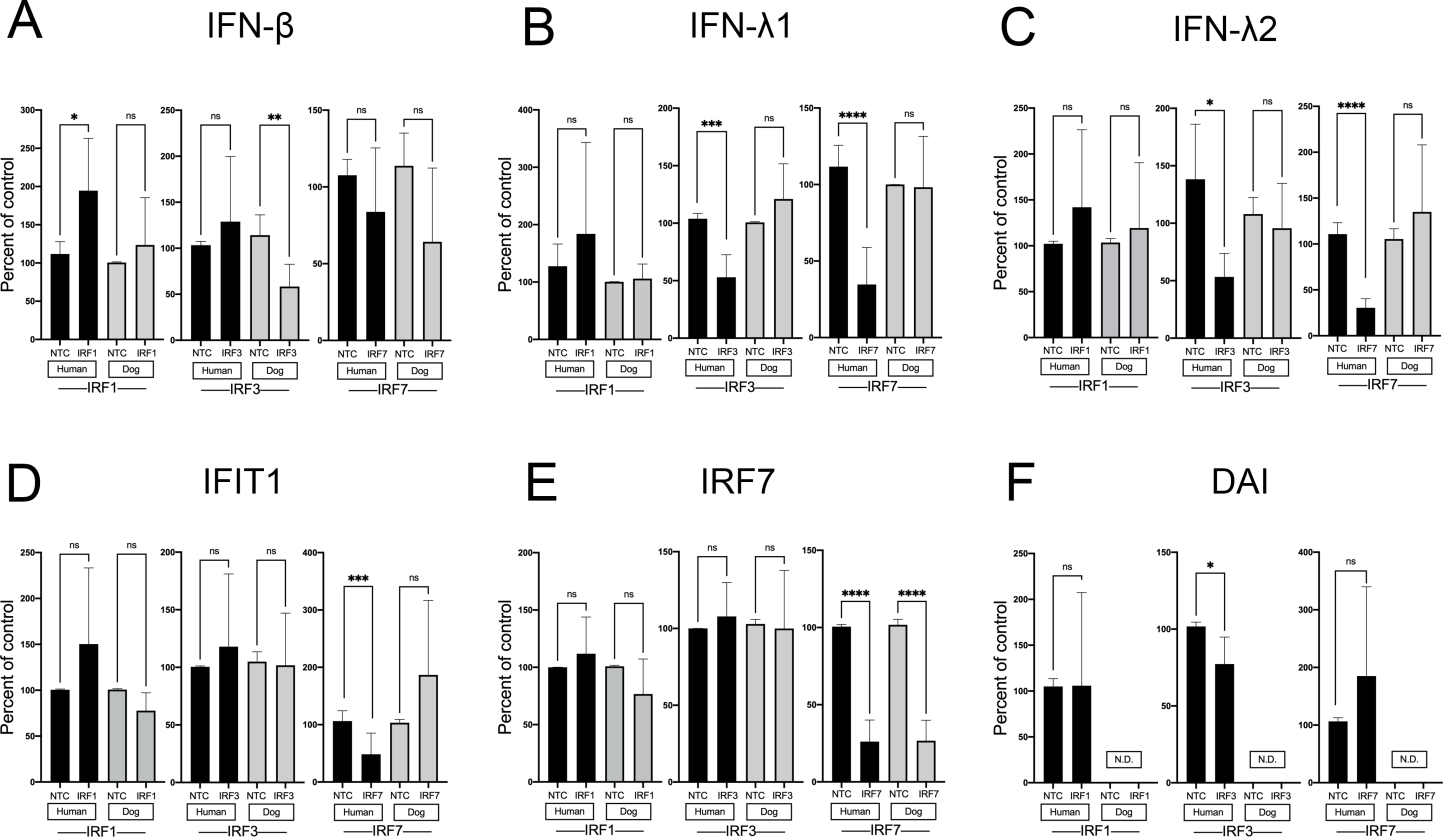
Basal expression of IFNs and ISGs in human and canine keratinocytes with knockout (KO) or knockdown (KD) of IRF1, IRF3, or IRF7. Human (hTERT) and canine (CPEK) keratinocytes were cultured for 24 hours before IFN and ISG expression levels were assessed by quantitative RT-PCR. Expression of IFN-β **(A)**, IFN-λ1 **(B)**, IFN-λ2 **(C)**, IFIT1 **(D)**, DAI **(E)** and IRF7 **(F)** in IRF1 KO, IRF3 KO, or IRF7 KD hTERTs and CPEKs are graphed as percent of non-targeting control (NTC) cells. Data represents the mean of 3–10 independent experiments performed in duplicate, ± SEM. Asterisks (*) indicate a significant difference (*p* < 0.05) in expression between NTC control and KO/KD cells, where *p < 0.05; **p < 0.01; ***p < 0.001; ****p < 0.0001. ns, not significant. N.D., not detected.

After creating stably silenced IRF1, IRF3, and IRF7 hTERT and CPEK cultures, we wanted to determine how the lack of these IRFs affected IFN and ISG expression basally. IRF1 had no significant effect on basal expression of any IFNs or ISGs in either hTERTs or CPEKs (**Figures 7 A–F**). In hTERTs, but not CPEKs, both IRF3 and IRF7 impacted IFN-λ1 (**Figure 7B**) and IFN-λ2 (**Figure 7C**) expression. In contrast, IRF3 significantly affected type I IFN expression in CPEKs but not hTERTs; IRF7 slightly impacted type I IFN expression in CPEKs, although this did not reach significance (**Figure 7A**).

### 3.8 IRF1 impacts early Poly(I:C) induced IFN and ISG expression in canine but not in human keratinocytes

**Figure 8:**
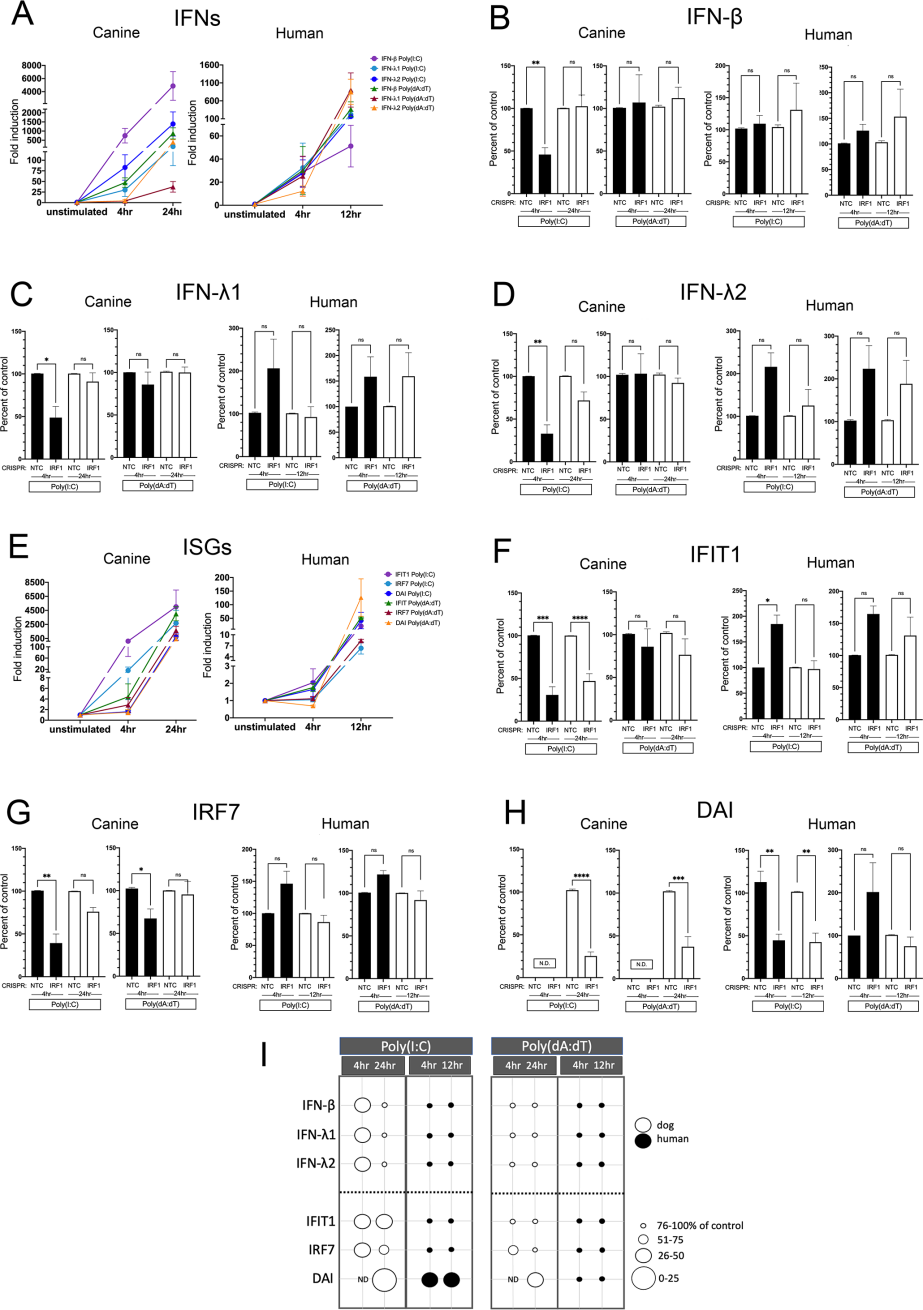
Induced expression of IFNs and ISGs in human and canine keratinocytes with knockout (KO) of IRF1. Non-targeting control (NTC) cells and IRF1 KO cells were assessed by quantitative RT-PCR for IFN and ISG expression levels 4 and 12 hours post stimulation with Poly(I:C) or Poly(dA:dT) in hTERTs, and after 4 and 24 hours post stimulation in CPEKs. Fold induction (ΔΔCt) of IFN **(A)** or ISG **(E)** mRNA expression in NTC CPEKs or hTERTs. Fold induction is graphed as expression in NTC cells over the unstimulated control (set to 1), and normalized to the reference gene, RPL13A. Expression of IFN-β **(B)**, IFN-λ1 **(C)**, IFN-λ2 **(D)**, IFIT1 **(F)**, IRF7 **(G)**, and DAI **(H)** in IRF1 KO hTERTs and CPEKs after Poly(I:C) or Poly(dA:dT) stimulation is graphed as the percent compared to NTC cells normalized to the reference gene RPL13A. Data represents the mean of 3 (dog) to 6 (human) independent experiments performed in duplicate, ± SEM. Asterisks (*) indicate a significant difference (*p* < 0.05) in expression between NTC and IRF1 KO cells, where *p < 0.05; **p < 0.01; ***p < 0.001; ****p < 0.0001 using one-way ANOVA statistical test. ns, not significant; N.D., not detected. **(I)** Bubble diagram summarizing the effect of IRF1 on expression of the IFNs and ISGs in human and canine keratinocytes. Bubble size indicates gene expression as an approximate percent of NTC cells- larger circles indicate a larger effect. Black circles, hTERTs; white circles, CPEKs. ND, not detected.

Fold induction of NTC cells at 4 and 12/24 hours for the IFNs and ISGs are shown in **Figures 8AE** respectively. As expected, early induction at 4 hours was lower in the downstream ISGs compared to the IFNs in both hTERTs and CPEKs. Interestingly, IRF1 had an early effect on all IFNs (**Figures 8B–D**) after Poly(I:C) stimulation in CPEKs, which was no longer evident at 24 hours, and had an early and later effect on ISG expression which was less prominent at peak induction at 24 hours (**Figures 8F–H**). In contrast, IRF1 KO in hTERTs had no effect on IFN or ISG expression with the exception of DAI (**Figure 8H**), which remained decreased at peak induction (12 hours) (**Figure 8H**). In CPEKs, IRF1 KO had no effect on any IFN expression (**Figures 8B–D**) after stimulation with Poly(dA:dT), but did significantly reduce DAI expression at 24 hours (**Figure 8H**). In hTERTs, IRF1 had no effect on IFN or ISG expression. An IFN-independent effect of IRF1 on DAI after Poly(I:C) stimulation in the dog could also be possible, however, this is less clear as IRF1 also affected early IFN expression which could account for the impact on DAI expression. In summary, IRF1 has a significant early impact on IFN expression after Poly(I:C) stimulation in canine keratinocytes, which likely results in an early and late reduction of downstream ISG expression. IRF1 in human keratinocytes after poly(I:C) stimulation impacted DAI expression only, most likely through an IFN-independent mechanism. After poly(dA:dT) stimulation, IRF1 only directly affected DAI in canine keratinocytes, potentially through an IFN-independent mechanism, and had no effect in human keratinocytes. Therefore, IRF1 differentially regulates IFNs, not only between species, but also between stimulus types. A summary diagram of the effect of IRF1 is shown in **Figure 8I**.

### 3.9 IRF3 is a major player in early and late IFN expression in both canine and human keratinocytes

**Figure 9:**
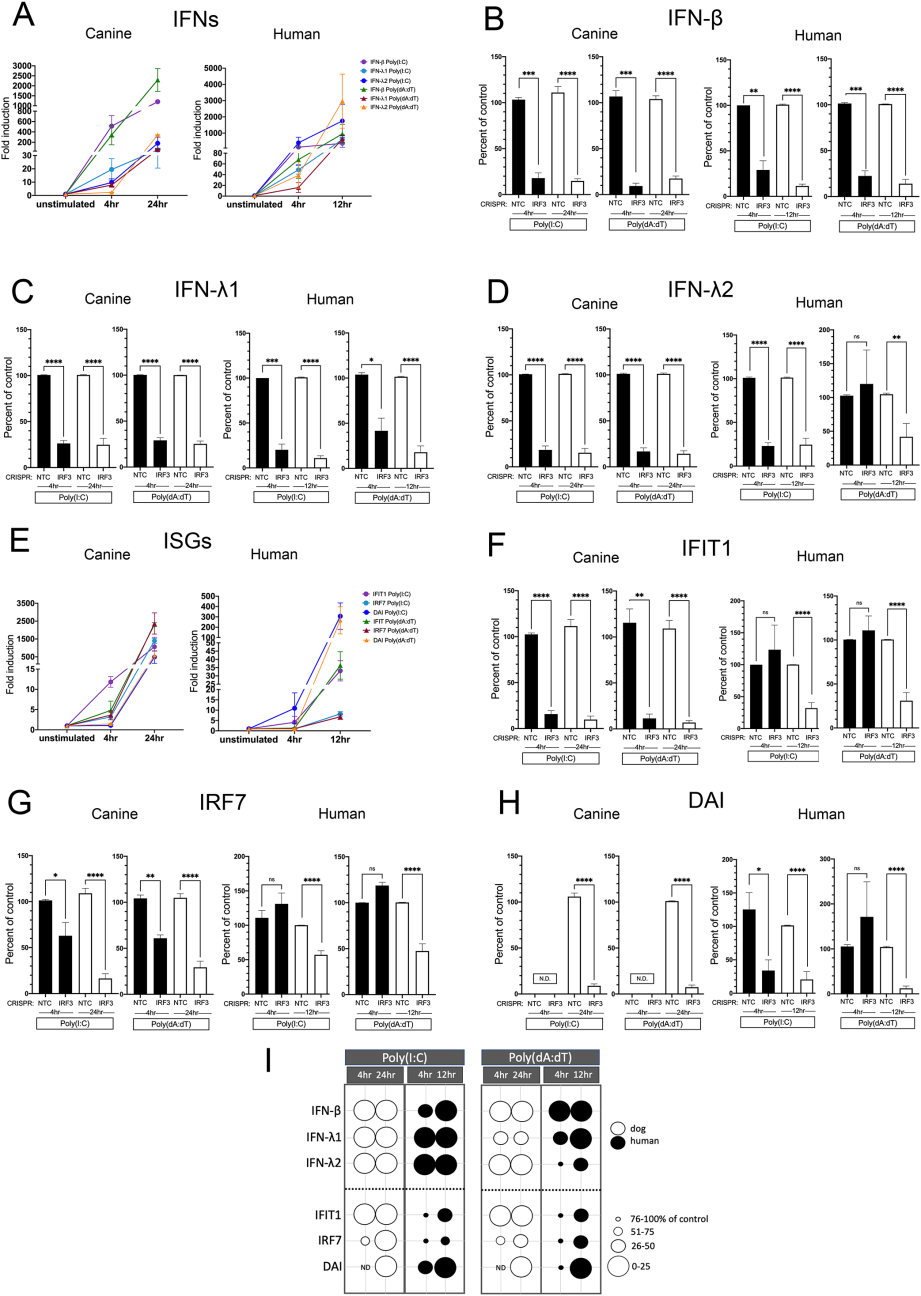
Induced expression of IFNs and ISGs in human and canine keratinocytes with knockout (KO) of IRF3. Non-targeting control (NTC) cells and IRF3 KO cells were assessed by quantitative RT-PCR for IFN and ISG expression levels 4 and 12 hours post stimulation with Poly(I:C) or Poly(dA:dT) in hTERTs, and after 4 and 24 hours post stimulation in CPEKs. Fold induction (ΔΔCt) of IFN **(A)** or ISG **(E)** mRNA expression in NTC CPEKs or hTERTs. Fold induction is graphed as expression in NTC cells over the unstimulated control (set to 1), and normalized to the reference gene, RPL13A. Expression of IFN-β **(B)**, IFN-λ1 **(C)**, IFN-λ2 **(D)**, IFIT1 **(F)**, IRF7 **(G)**, and DAI **(H)** in IRF3 KO hTERTs and CPEKs after Poly(I:C) or Poly(dA:dT) stimulation is graphed as the percent compared to NTC cells normalized to the reference gene RPL13A. Data represents the mean of 3 (dog) to 6 (human) independent experiments performed in duplicate, ± SEM. Asterisks (*) indicate a significant difference (*p* < 0.05) in expression between NTC control and IRF3 KO cells, where *p < 0.05; **p < 0.01; ***p < 0.001; ****p < 0.0001 using one-way ANOVA statistical test. ns, not significant. N.D., not detected. **(I)** Bubble diagram summarizing the effect of IRF3 on expression of the IFNs and ISGs in human and canine keratinocytes. Bubble size indicates gene expression as an approximate percent of NTC cells- larger circles indicate a larger effect. Black circles, hTERTs; white circles, CPEKs. ND, not detected.

Fold induction of NTC cells at 4 and 12/24 hours for the IFNs and ISGs are shown in **Figures 9A–E** respectively. Not surprisingly, IRF3 had a significant and sustained impact on all IFN expression at 4 and at 12/24 hours in both human and canine keratinocytes (**Figures 9B–D**) after stimulation with Poly(I:C). Stimulation with Poly(dA:dT) resulted in a significant and sustained impact on expression of all IFNs at 4 and 12/24 hours in both canine and human keratinocytes with the exception of human IFN-λ2, which was not affected until 12 hours after stimulation. In canine keratinocytes, IRF3 KO caused an early sustained reduction in ISG expression after both Poly(I:C) and Poly(dA:dT) stimulation (**Figures 9F–H**). In hTERTs, IRF3 KO caused a late reduction in ISG expression only at 12 hours post stimulation with either Poly(I:C) or Poly(dA:dT), apart from the early decrease seen in DAI after Poly(I:C) stimulation (**Figures 9F–H**). A summary diagram of the effect of IRF3 is shown in **Figure 9I**.

### 3.10 IRF7 impacts IFN expression in both canine and human keratinocytes

**Figure 10:**
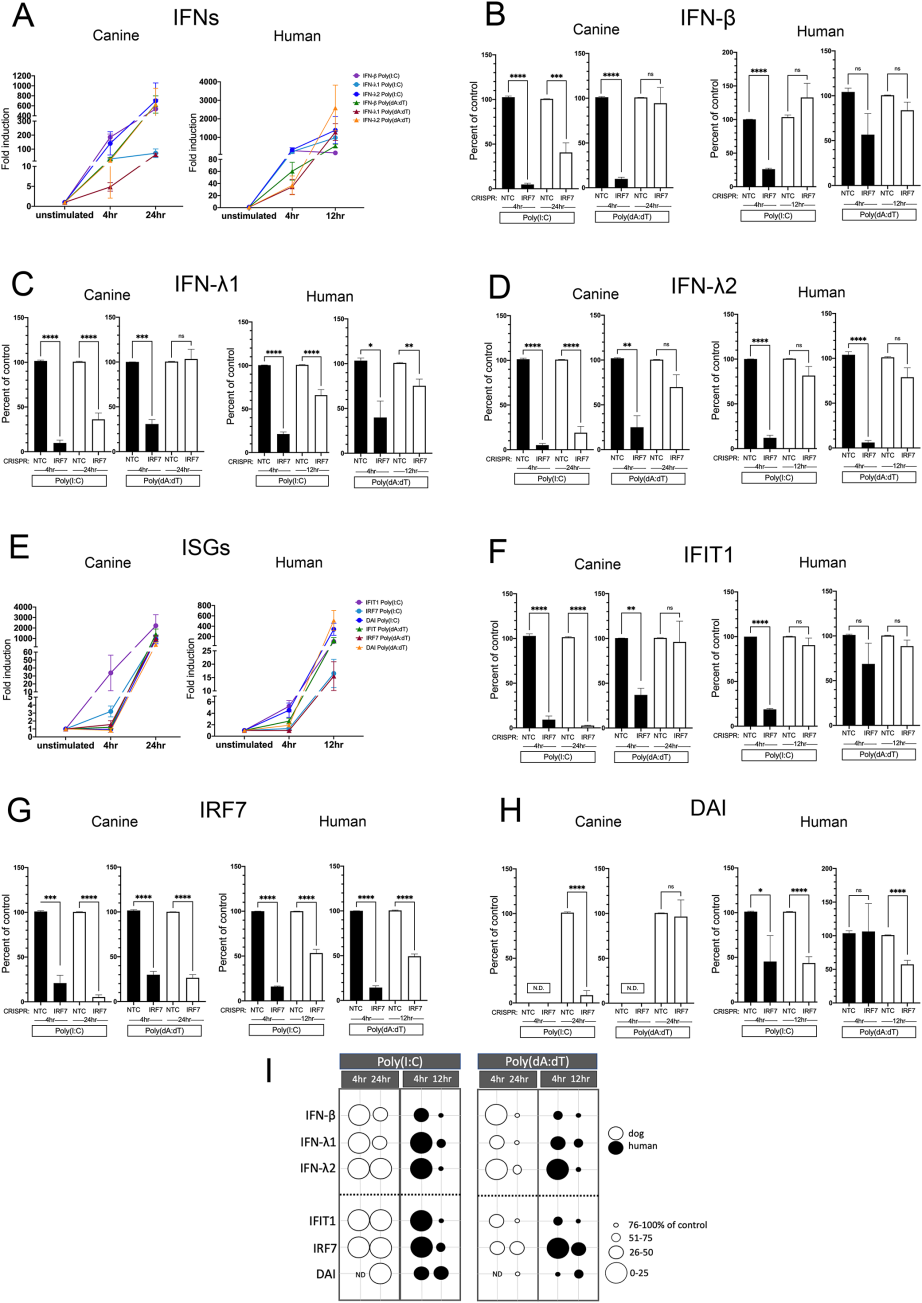
Induced expression of IFNs and ISGs in human and canine keratinocytes with knockdown (KD) of IRF7. Non-targeting control (NTC) cells and IRF7 KD cells were assessed by quantitative RT-PCR for IFN and ISG expression levels 4 and 12 hours post stimulation with Poly(I:C) or Poly(dA:dT) in hTERTs, and after 4 and 24 hours post stimulation in CPEKs. Fold induction (ΔΔCt) of IFN **(A)** or ISG **(E)** mRNA expression in NTC CPEKs or hTERTs. Fold induction is graphed as expression in NTC cells over the unstimulated control (set to 1), and normalized to the reference gene, RPL13A. Expression of IFN-β **(B)**, IFN-λ1 **(C)**, IFN-λ2 **(D)**, IFIT1 **(F)**, IRF7 **(G)**, and DAI **(H)** in IRF7 KD hTERTs and CPEKs after Poly(I:C) or Poly(dA:dT) stimulation is graphed as the percent compared to NTC cells normalized to the reference gene RPL13A. Data represents the mean of 3 (dog) or 6 (human) independent experiments performed in duplicate, ± SEM. Asterisks (*) indicate a significant difference (*p* < 0.05) in expression between NTC control and IRF7 KD cells, where *p < 0.05; **p < 0.01; ***p < 0.001; ****p < 0.0001 using one-way ANOVA statistical test. ns, not significant. N.D., not detected. **(I)** Bubble diagram summarizing the effect of IRF7 on expression of the IFNs and ISGs in human and canine keratinocytes. Bubble size indicates gene expression as an approximate percent of NTC cells- larger circles indicate a larger effect. Black circles, hTERTs; white circles, CPEKs. ND, not detected.

Fold induction of shNTC control cells at 4 and 12/24 hours for the IFNs and ISGs are shown in **Figures 10A–E** respectively. After Poly(I:C) stimulation in CPEKs, IRF7 had a significant early effect on all IFNs which is sustained through peak induction at 24 hours (**Figures 10B–D**), as well as an early reduction of ISG expression which is sustained through peak induction at 24 hours (**Figures 10F, H**). After Poly(I:C) stimulation in hTERTs, IRF7 had a similar early effect on IFNs but was no longer evident by 12 hours (**Figures 10B, D**), and a significant early decrease in ISG expression which was sustained at 12 hours in DAI but not IFIT1 (**Figures 10F, H**). Results were similar after Poly(dA:dT) stimulation of CPEKs: IRF7 had a significant early effect on all IFNs which was no longer evident at 24 hours (**Figures 10B–D**) and an early effect on IFIT1 expression which was also not seen by 24 hours. After Poly(I:C) stimulation in hTERTs, IRF7 had an early effect on IFN expression, although the impact on type I IFN did not reach significance, and expression was mostly recovered by 12 hours. The ISGs IFIT1 and DAI also had early decreased expression, which was significantly sustained at 12 hours in DAI but not in IFIT1 (**Figures 10F, H**). In summary, IRF7 affected IFNs early in both CPEKs and hTERTs after stimulation with both Poly(I:C) and poly(dA:dT), which was sustained in the CPEKs after poly(I:C) stimulation only, and in hTERT IFN-λ1 after stimulation with both ligands. ISG expression was also reduced early by IRF7 in both CPEKs and hTERTs after poly(I:C) and in IFIT1 in CPEKs after poly(dA:dT). IRF7 knockdown in both cell lines was verified by mRNA expression. IRF7 mRNA levels were reduced by approximately 75-90% in CPEKs, and by 50-85% in hTERTs (**Figure 10G**). A summary diagram of the effect of IRF7 is shown in **Figure 10I**.

The early impact of IRF7 on both IFN and ISG expression in both human and canine keratinocytes was unexpected, as IRF7 is often considered part of the secondary response after sufficient IRF7 is upregulated after initial stimulation. Based on these results, it suggests that basal levels of IRF7 are sufficient to have an early impact on IFN upregulation. Because of these unexpected results, we wanted to determine if this finding could be observed in non-keratinocyte cells. We chose two cell lines derived from kidney, MDCK which are canine cells and HEK293 which are human cells. IRF7 knockdown was performed using the same shRNAs as used for the CPEKs and hTERTs, which were then stimulated with poly(I:C) and poly(dA:dT) as above. Cells were lysed for IFN and ISG gene expression analysis after 4 hours and 24 hours as described above. Fold induction of shNTC control cells at 4 and 24 hours for the IFNs and ISGs are shown in **Supplementary Figures 3A, E** respectively. Results demonstrated that IRF7 knockdown in HEK293 has minimal impact on IFN or ISG expression at any timepoint (**Supplementary Figures 3B–D, F–H**). This lack of response in HEK293 cells may be due to the not strictly epithelial origin of this cell line. MDCKs, however, demonstrated an early impact on all IFNs which was similar to response seen in CPEKs (**Supplementary Figures 3B–D**). This response was sustained longer (impact evident at both 4 and 24 hours) in MDCKs compared to CPEKs, which was particularly apparent when stimulated with poly(dA:dT) (**Supplementary Figures 3B–D**). A similar pattern was noted for IFIT1 (**Supplementary Figure 3F**). DAI was not upregulated after stimulation with either ligand (**Supplementary Figure 3H**). These results suggest that IRF7 can play a role in the early induction of IFN and ISG in multiple cell types.

## 4 Discussion

Regulation of type I and III interferons, which are critical in antiviral defense, are often cell-type specific; and, while much is known in regards to regulation of the interferon response in many cell types, little is known regarding regulation of the type I and III interferons in keratinocytes. Thus, we sought to discern the expression kinetics of type I and III IFN in response to cytosolic dsDNA and dsRNA, and how this response is regulated by IRF1, IRF3, and IRF7, the major IRFs responsible for upregulation of type I and III IFNs in other epithelial cell types (32–35). We compared the response between human and canine keratinocytes, which would advance the dog as a large animal model for studying innate antiviral immunity and also uncover potential species-specific differences. We found that while type III interferons dominated the human IFN response to both cytosolic dsDNA and dsRNA compared to type I, both type III and type I interferons were comparably expressed in the dog, with a higher induction of type I over type III interferons, likely due to lower basal expression of type I IFNs. Human keratinocytes, on the other hand, were pre-armed at baseline with higher basal expression of type I IFNs and higher ISGs, including basal IRF7, suggesting that there is a protective effect against viruses or other pathogens. This high basal type I IFN and ISGs also resulted in a more blunted type I IFN and ISG induced response in human keratinocytes. Regulation of the antiviral response to preferentially induce type I versus type III was not due to differential activation of IRFs, as both IRF3 and IRF7 significantly and similarly impacted both type I and III IFN expression in dog and human keratinocytes; IRF1 impacted only early type I and III IFN expression in dog keratinocytes in response to dsRNA.

### 4.1 Basal IFN and ISG expression

Human keratinocytes expressed higher copies of basal mRNA type I (IFN-β), whereas dog keratinocytes expressed higher copies of basal type III IFNs (**Figure 3**). Additionally, human keratinocytes had significantly higher basal mRNA expression of IRF7 and IFIT1 compared to dog keratinocytes, which had lower basal ISGs. This indicates transcription of these important ISGs is occurring basally in human keratinocytes; as IFN-β is a known regulator of ISGs, the higher basal IFN-β expression in human keratinocytes may account for higher ISG expression. Since knockdown of IRF7 in human keratinocytes did not impact IFN-β expression (**Figure 7**), the high basal IRF7 is not driving basal IFN-β expression. In fact, none of the IRFs we examined impacted basal IFN-β expression in human keratinocytes, and there is likely different, 1, -3, or -7, mechanisms driving basal IFN-β expression in human keratinocytes. It should be noted that one of the limitations of this study is the incomplete knockdown or knockout of the IRFs, and it is unlikely, but possible, that this small remaining IRF expression is enough to drive basal IFN expression. However, mechanisms leading to basal IFN expression would be consistent with previous studies which have shown that in other cell types, human basal IFN-β is regulated in an IRF-independent manner, and relies more upon c-Jun and NF-κB components (7). Future knockout studies targeting individual AP1 and NF-κB components would help to identify key regulators of basal IFN expression in keratinocytes. Although basal IRF7 does not impact basal IFN-β expression, our knockdown studies in human keratinocytes determined that basal IRF7 does affect basal IFIT1, and the high basal IRF7 likely contributes to the high basal IFIT1 expression in human keratinocytes. While none of the IRFs we examined impacted basal IFN-β expression in human keratinocytes, IRF3 had a partial, but significant impact on IFN-β expression in dog keratinocytes. As basal IFN-β expression was already very low in the dog, the biological significance of this small impact is unknown. Basal type III IFNs was higher in dog compared to human keratinocytes and this expression was not driven by any of the IRFs we examined, putatively suggesting a non-IRF1, -3, or -7, mechanism driving basal type III expression in dog keratinocytes, such as NF-κB, as suggested in previous studies (39). Interestingly, while none of the IRFs we examined impacted basal type III expression in dog keratinocytes, both IRF3 and IRF7 impacted type III expression in human keratinocytes. While basal type III expression was already very low in human keratinocytes, the biological significance of this small impact of IRF3 and IRF7 on its expression is unknown. Thus, the higher basal expression of the dominant IFN in human and dog keratinocytes, type I and type III respectively, is putatively driven by non-IRF1, -3, or -7, mechanisms. Understanding these mechanisms underlying basal type I and type III IFN, and ultimately basal ISG expression, could have significant implications for modulating the immune response to pathogens. In cardiac myocytes, for example, high basal expression of IFN-β resulted in higher basal ISG (IRF7 and IFIT1) expression, and greater protection from viral infections in the absence of IFN than cardiac fibroblasts, which had lower basal IFN-β and ISGs and depended more on the viral-induced IFN response (40). Additional studies using functional assays, such as viral infectivity and viral replication assays, would help to demonstrate how these differences in basal IFN and ISG expression could protect from viral infections, and if this protection was lost with inhibition of basal IFN or ISG expression.

Interestingly, even though dog keratinocytes had higher basal type III IFNs than human, this did not result in high basal ISGs expression. This suggests that despite both type I and III IFNs pathways activating the Jak-Stat pathway, there are additional mechanisms regulating ISG expression differentially by type I versus III IFNs. Further, there is differential activation of the ISGs generally, for example, IRF7 and IFIT1 are elevated basally in human keratinocytes while DAI is barely expressed. Since ISGs are upregulated by the Jak-Stat pathway in response to both type I and III IFNs, there are additional regulatory mechanisms that guide and tailor the ISG response. Regulation of the ISG response between IFN-α and IFN-β, for example, which both signal through the same receptor, is maintained by different binding affinities for their receptor between these two IFNs (41). It is possible, therefore, that similar mechanisms exist to help guide the ISG response between type I and III IFNs, even though they signal through different receptors.

While only a small number of ISGs were examined, the higher basal expression of IFN-β and ISGs in human keratinocytes compared to dog suggests that human keratinocytes are pre-armed for an antiviral response. In many cell types, ISGs including IRF7 are only present basally at low levels prior to stimulation, likely to minimize potential aberrant inflammation-inducing signaling. However, the sheer location of keratinocytes on the skin surface exposes them to many more pathogens than other cell types, such as pulmonary epithelial cells. As such, pre-arming of the antiviral response could enable them to respond early to pathogens or prevent infection altogether. ISGs can target every stage of the viral life cycle, and some, like IFIT1, have been shown to have multiple antiviral functions, such as viral RNA degradation, or inhibition of viral replication or protein translation (42). More specifically, for example, IFIT1 binds to the E1 viral early gene of papillomavirus, inhibiting its ability to initiate viral replication (43), thus cells with high levels of basal IFIT1 could prevent infection earlier than cells with lower basal amounts. However, there must be a balance so that adequate pathogen control does not lead to immune dysregulation. Keratinocytes are likely able to tolerate these higher basal IFNs and ISGs, as they interact with very few immune type cells that reside within the epidermis. High basal levels of inflammatory mediators in other epithelial cells, such as pulmonary epithelial cells, could result in aberrant inflammation at vulnerable sites, where inflammation in the lung can lead to loss of air-exchange within alveoli. We hypothesize that the high basal type I IFN and ISGs enables a faster or more robust immune response to frequent pathogen exposure in human skin, and that the presence of dog fur may decrease pathogen exposure to dog keratinocytes, and thus they do not have to be as pre-armed for an antiviral immune response. The only other known cell types to express high basal type I IFNs and/or ISGs are non-dividing cells, including neurons of the hippocampus and cardiac myocytes (44, 45). Hippocampal neurons exhibited high basal type I IFNs but lower select basal ISGs. In this context however, the high basal type I IFNs was sufficient to control the infection early in absence of exogenous type I IFN. Further, in support of our findings, ISGs with low basal levels were more highly upregulated after IFN-β induction (44). Cardiac myocytes also expressed higher basal type I IFNs but in contrast to hippocampal neurons, and in support of our data, also expressed higher levels of a subset of ISGs including IRF7, which the authors attributed to the high basal type I IFN (45). These papers suggest that high basal type I IFNs then play a role in protection of susceptible non-dividing cells. However, unlike cardiac myocytes or neurons, keratinocytes are an actively dividing cell population, thus ultimately, ISG responsiveness to higher basal type I IFNs may be regulated in a cell-type and/or dividing versus non-dividing specific manner. In keratinocytes, it may be the higher frequency for pathogen exposure that warrants this “priming” of keratinocytes in human. Further studies could help discern significant differences on the impact of high basal IFN and ISG expression in non-dividing versus proliferating cells. Even within keratinocytes, differences between basal IFN and ISG expression could be compared between a proliferating basal cell compared to a keratinocyte that has been induced to differentiate, where it has entered a non-proliferating state. Further, the dog could be used to help elucidate mechanisms underlying expression of basal type I IFNs and ISGs in human keratinocytes, and how this priming can ultimately affect their response to viruses and other pathogens.

Basal expression of IFNs and ISGs significantly impacted the upregulation of these genes after stimulation with cytosolic dsDNA and dsRNA. For humans, high basal type I IFN, IFIT1, and IRF7 resulted in their blunted upregulation when compared with the marked upregulation of type III IFNs and DAI. Ultimately, though, stimulation with cytosolic dsDNA and dsRNA resulted in high copy numbers of all IFNs and ISGs in human keratinocytes, with significantly higher type III IFNs than type I IFNs. This observation agrees with previous studies that have shown that the type III IFN response predominates in epithelial cells after stimulation (30, 46). In contrast, the lower basal expression of type I IFN, IFIT1, and IRF7 in dog keratinocytes resulted in marked upregulation of these genes after stimulation, greater than seen in human keratinocytes. The upregulation of type III IFNs was more blunted as dog keratinocytes had higher basal type III IFN expression, and ultimately there was only slightly higher copies of type III IFNs compared with type I IFNs at peak upregulation. Thus, the species-specific differences in the basal levels of IFNs and ISGs impact the differences in induced IFNs and ISGs. Ultimately, though, total copy number of all IFNs and ISGs was always higher in human keratinocytes than dog keratinocytes, regardless of basal levels and degree of induction. This indicates that human keratinocytes have a higher threshold limit for the abundance of IFNs or ISGs that can be produced. We postulate that this higher threshold limit in human keratinocytes could be the result of higher basal IRF7, allowing IRF7 to heterodimerize with IRF3 immediately after activation, resulting in rapid and robust IFN expression. Previous studies have shown that heterodimerization of IRF3 and IRF7 may be more critical for early IFN induction (3), and dog keratinocytes, with little basal IRF7, may rely more on IRF3 homodimerization for the initial IFN response. Future co-immunoprecipitation and chromatin immunoprecipitation experiments could help to determine the significance of IRF3 versus IRF7 heterodimers and homodimers.

### 4.2 Type I and III IFN induction

Previous studies have shown that induction of type III IFN occurs more slowly than type I IFNs in epithelial cells (2, 5). In both dog and human keratinocytes, however, peak upregulation of type I and type III IFNs occurred concurrently, suggesting that one IFN type does not work faster, or induce the other. Other studies support our findings, where in pulmonary epithelial cells the upregulation kinetics between type I and type III were similar but their relative levels of mRNA induction differed (47). Thus, the kinetics of type I versus type III IFN upregulation seem to be cell-type specific, and for both dog and human keratinocytes, type I and III IFNs peak concurrently after stimulation with cytosolic dsDNA and dsRNA. Human keratinocytes reached peak upregulation of IFNs consistently at 12 hours, which was faster than peak upregulation in dog keratinocytes, which occurred on average about 24 hours post stimulation. This faster response of human keratinocytes could be due, in part, to the “priming” of high levels of basal IRF7 and other ISGs. While dog keratinocytes had higher levels of basal IRF3, basal IRF3 has limited function without stimulation, and heterodimerization between IRF3 and IRF7 only occurs after sufficient IRF7 is produced.

### 4.3 Impact of IRF3 on IFN and ISG response

IRF3 is found in most cell types, and is one of the main IRFs responsible, along with IRF7, for type I and III IFN expression, upon activation by viral infection (48, 49). Thus, not surprisingly, we found that both human and dog keratinocytes rely significantly on IRF3 for IFN and ISG expression after stimulation with cytosolic dsDNA and dsRNA. Interestingly, IRF3 had a significant impact on ISGs early after stimulation in dog keratinocytes when compared with human ISGs. We speculate that although dog keratinocytes take longer for IFN induction compared to human keratinocytes, they may have faster initial induction of the ISGs after ligand stimulation, likely to compensate for the lower ISGs basally. Along these lines, it is possible that some ISGs are rapidly upregulated early due to a direct IFN-independent IRF3-mediated induction. Previous studies have shown that IRF3 can directly upregulate IFIT1 in the absence of IFN and may also directly interact with DAI (39, 40).

### 4.4 Impact of IRF1 on IFN and ISG response

IRF1 is critical for the antiviral response, particularly for the type III IFN response in certain cell types (1). IRF1 has been shown to have multiple functions in addition to induction of IFNs and ISGs, such as tumor suppression, apoptosis, and induction of pro-inflammatory cytokines (34). The mechanistic functions of IRF1 in IFN induction are complex and unclear, as it has been shown to be involved in both type I and type III IFN-mediated and IFN-independent ISG induction in different contexts, within different cell types (33). IRF1 was shown to have a critical role in the early (6hr) but not late (24hr) type I and III IFN response to dsRNA in human respiratory epithelial cells (33). Interestingly, we found that IRF1 had no impact on IFN or ISG expression in human keratinocytes in response to either cytosolic dsDNA or dsRNA. In contrast, IRF1 in dog keratinocytes had a significant impact on early (4hr) but not late (24hr) IFN induction after stimulation with cytosolic dsRNA but not dsDNA. This result is similar to that seen in human respiratory epithelial cells, but not human keratinocytes, and was limited to dsRNA. Thus, the effect of IRF1 appears to be stimulus specific, as well as cell type and species specific. We speculate that human respiratory epithelial cells, like dog keratinocytes, may exhibit low basal ISGs to prevent aberrant inflammation, and that IRF1 compensates for low basal ISGs by enhancing the initial early upregulation of IFNs and ISGs, either by augmenting the activation of IRF3 (34), or by direct binding of IRF1 to IFN promoters.

In dog keratinocytes, IRF1 was also crucial for upregulation of all the ISGs both early and at 24 hours. This early effect of IRF1 on the ISGs, like IRF3, supports the possibility that although peak induction of IFNs in dog keratinocytes may occur later than in humans, the early generation of ISGs may occur more rapidly, either through IFN receptor-mediated signaling, where canine IFNs could have a higher affinity for IFN receptors than human IFNs, or directly by the IRFs themselves. Alternatively, dog keratinocytes may have an increased number of type I IFN receptors compared to human, allowing for an earlier robust upregulation of ISGs to compensate for the lower ISGs basally. IRF1 has been shown to directly upregulate DAI through IFN-independent mechanisms (50), which could also explain why in human keratinocytes, the only effect of IRF1 was through an IFN-independent direct induction of DAI after cytosolic dsRNA stimulation. In humans, previous studies have shown that IRF1 is most strongly induced by IFN-γ signaling and upregulates IFNs through TLR mediated signaling (3). Thus, it is possible that IRF1 may just be poorly activated by cytosolic sensors in human keratinocytes, but more strongly activated by cytosolic sensors in dogs.

### 4.5 Impact of IRF7 on IFN and ISG response

We demonstrate that in both dog and human keratinocytes, IRF7 has an early effect on both type I and III IFN and ISG induction. The canonical IFN pathway generally maintains that due to the continuous production of IRF7 through the feedback loop, IRF7 is more necessary later in infection for sustained IFN production, while IRF3 is degraded (51). However, we showed that IRF7 was important in the immediate early response after stimulation. As this was somewhat unexpected, we also tested non-keratinocyte cell lines and found a similar result in the canine epithelial cells. While broad conclusions regarding this early IRF7 impact in non-keratinocytes is not possible, it does suggest that it is not a unique keratinocyte finding. While in human keratinocytes this could be explained by the presence of the high levels of basal IRF7, this same immediate early response requiring IRF7 was also seen in dog keratinocytes with lower basal IRF7. It is possible that mRNA expression does not correlate with IRF7 protein expression, and both human and dog keratinocytes could maintain the same basal IRF7 protein levels. IRF7 is generally considered to have a short half-life and is heavily regulated at the protein level; however, there are known cell-type specific differences in the half-life of IRF7, and it is possible that basal IRF7 protein is maintained in both dog and human keratinocytes to be used early in an antiviral response (52). It is also possible that dog keratinocytes have low basal IRF7 protein, and the effect of IRF7 seen at 4 hours post-stimulation reflects newly synthesized IRF7 and not basal IRF7. While this early effect was somewhat surprising, this is supported by numerous studies and reviews, and interestingly, was found in other species as well (3, 5, 52, 53).

**Figure 11:**
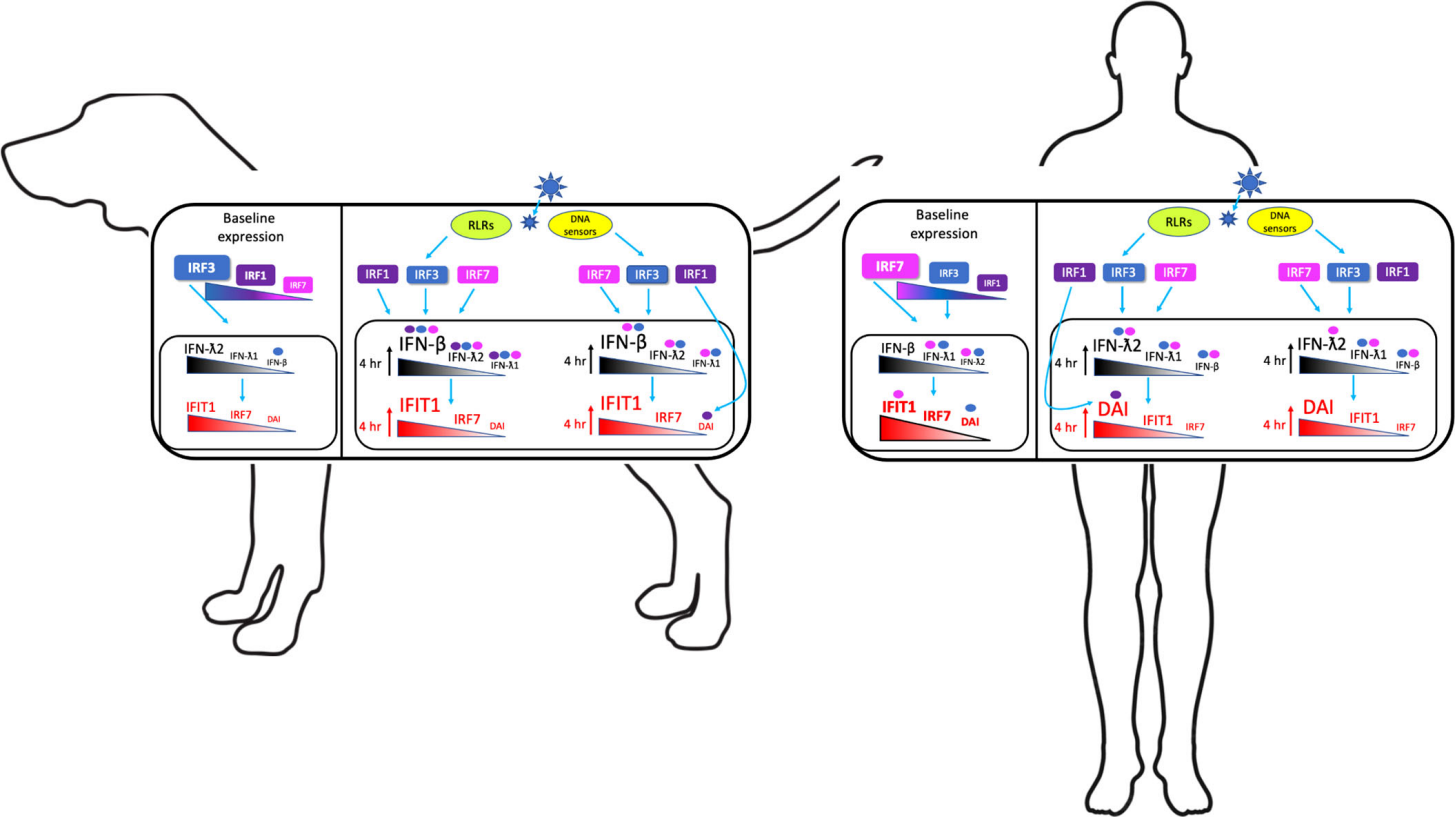
Schematic summary of IRF and basal and induced IFN and ISG expression in human and canine keratinocytes. Interferon regulatory factors (IRFs), type I and III interferon (IFN), and IFN stimulated gene (ISG) expression from highest to lowest are indicated by the triangular wedge, as well as by font or rectangle size. Blue arrows indicate flow of the signaling pathway. The Poly(I:C) ligand triggered the RLRs, or cytosolic RNA sensors, while the Poly(dA:dT) ligand triggered the cytosolic DNA sensors to initiate signaling to activate IRFs. The impact of IRF1 on IFN or ISG induction at 4 hours post-stimulation (as shown by a black or red up arrow) is indicated by a purple circle. The impact of IRF3 or IRF7 on IFN or ISG induction at 4 hours is indicated by a blue or pink circle, respectively.

It was also somewhat unexpected that IRF7, and for that matter IRF1, did not favor expression of either type I versus type III IFN expression. IRF1 in other cell types has been shown to be required for production of type III but not type I IFN, while the IFN-λ2 promoter has been shown to have a high affinity for IRF7 (1, 39). Thus, the regulation of a predominately type I versus a type III IFN response does not seem to be directly influenced by differential IRF 1, 3, or 7 activation. A schematic diagram of IRF expression, and their impact on basal and induced IFN and ISG expression is summarized in **Figure 11**. The differences in basal, and induced type I versus type III IFN expression may be driven by other IRF-independent mechanisms, such as NF- κB-mediated regulation. As stated above, it should be noted that one of the limitations of this study is the incomplete knockdown or knockout of the IRFs, and thus it remains possible that only a small amount of IRF expression is required to differentially regulate the type I versus type III response, and that incomplete knockdown or knockout of IRFs would not be sufficient to identify an impact. Additional experiments using IFN receptor, adaptor protein, and other transcription factor knockout cells could help decipher IRF-independent and species-specific mechanisms for differential upregulation of type I versus type III IFNs as well as IFN-dependent versus independent ISG upregulation in keratinocytes.

We tried to address potential differences between dog and human cell lines by examining the viral immune response in a variety of different cell lines obtained from different sites and from different sources and by adapting canine cells to human cell culture media to try to address if these differences accounted for the differences seen in the immune response between human and dog keratinocytes. Results from these experiments yielded similar results to those seen with the initial cell lines, suggesting that the differences in the type I versus type III upregulation are likely true differences between human and dog keratinocytes.

### 4.6 Comparative skin immunology

There is no universal animal model for studying innate IFN immunity and the associated diseases in human skin, as one may be more relevant than others depending on the disease. Pig skin is considered the closest to human skin histologically and morphologically (54); however, pigs are not known to spontaneously develop some of the same immunologically-associated skin disorders as humans and are not exposed to the same environmental factors. Dogs, however, do develop spontaneous immunologically-associated skin disorders which are similar to humans, and are exposed to the same environment. Dogs have been used as a successful animal model to study skin disorders such as atopic dermatitis, systemic lupus erythematosus, pemphigus foliaceous, and dermatomyositis, which can all be driven by a dysregulated IFN system (16, 19, 21, 55). In addition, an immunodeficient colony of dogs has been used as a model for cutaneous papillomaviruses (18). Moreover, dogs share >70% homology to human IFN genes and >80% homology with human IRFs (25), a greater degree of similarity to human than the mouse, a common animal model for studying innate immunity and disease.

### 4.7 Summary

We have demonstrated that there are some species-specific differences in human and canine keratinocytes between IRF-mediated type I and type III IFN regulation and the ISG response which would need to be taken into account when using the dog as a model for IFN-associated diseases and disorders. However, discerning these differences has identified the dog’s potential for studying the underlying mechanisms between the type I and type III IFN pathways, and future studies can answer questions regarding how keratinocytes direct regulation in favor of type I versus type III basal or induced IFN expression, or how human keratinocytes maintain basal type I and ISG expression, and their implications on IFN-dependent versus IFN-independent ISG antiviral responses. Defining the mechanistic differences between regulation of type I and type III IFNs and the ISG response is particularly relevant as currently, type I IFNs are used as therapies for several viral diseases, neoplasms and immune-mediated disorders in both humans and dogs, but due to their more systemic pro-inflammatory side effects, type III IFNs are being investigated as an alternative treatment which would result in less toxicity. Thus, using the dog as a model to unravel details of the mechanistic properties between type I and type III IFNs in dogs and humans can help advance the IFNs potential as treatment strategies.

## Data Availability

The raw data supporting the conclusions of this article will be made available by the authors, without undue reservation.
