# Supplementary material for: Comparative analysis of the impact of interferon regulatory factors on basal and induced interferon and interferon stimulated gene expression in human and canine keratinocytes
Source: Front Immunol. 2026 May 15;17:1810139. doi: 10.3389/fimmu.2026.1810139 (PMC13218971; doi:10.3389/fimmu.2026.1810139)
Supplement: Supplementary file 1 [file DataSheet1.pdf]

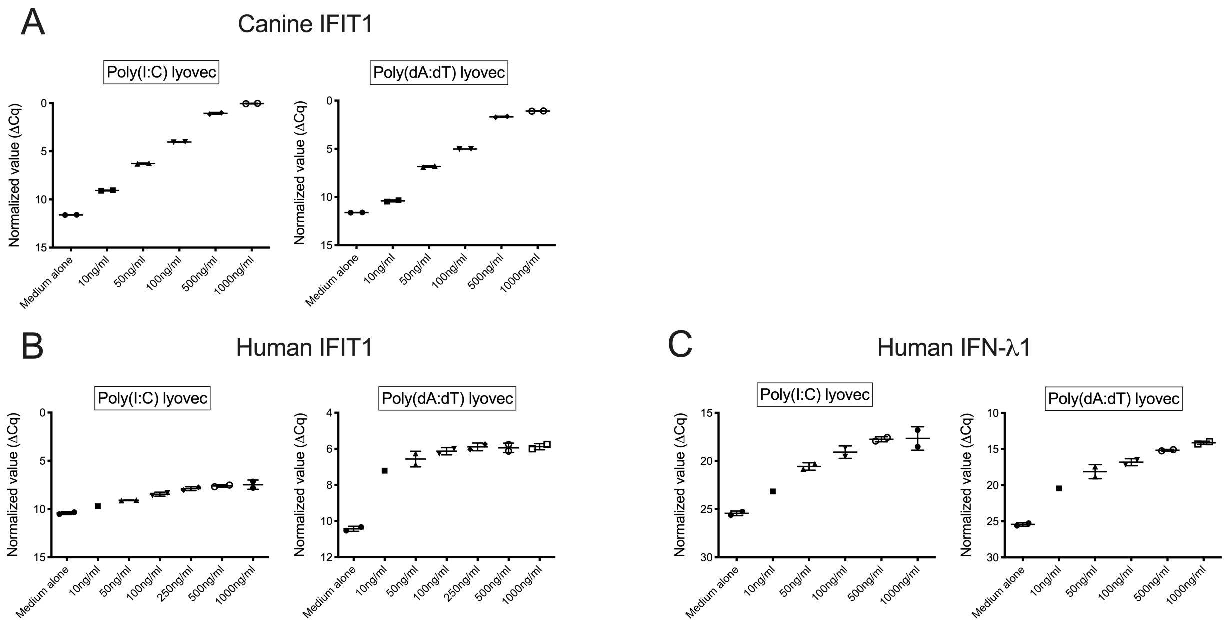

Supplemental Figure 1. Poly(I:C) and Poly(dA:dT) dose response curve in canine and human keratinocytes. Cultured canine CPEKs and human NHEKs were mock stimulated, or stimulated with 10ng/ml up to 1000ng/ml Poly(I:C) and Poly(dA:dT) for 24 hours before mRNA expression of IFIT1 and IFN-λ1 was assessed by quantitative RT-PCR. Expression levels of IFIT1 in CPEKs **(A)**, IFIT1 in NHEKs **(B)**, and IFN-λ1 in NHEKs **(C)** are graphed as the Delta Cq normalized to the RPL13A reference gene. The Y axis is reversed to position higher expression levels higher on the graph. Data represents a single experiment performed in duplicate, ± standard deviation (SD).

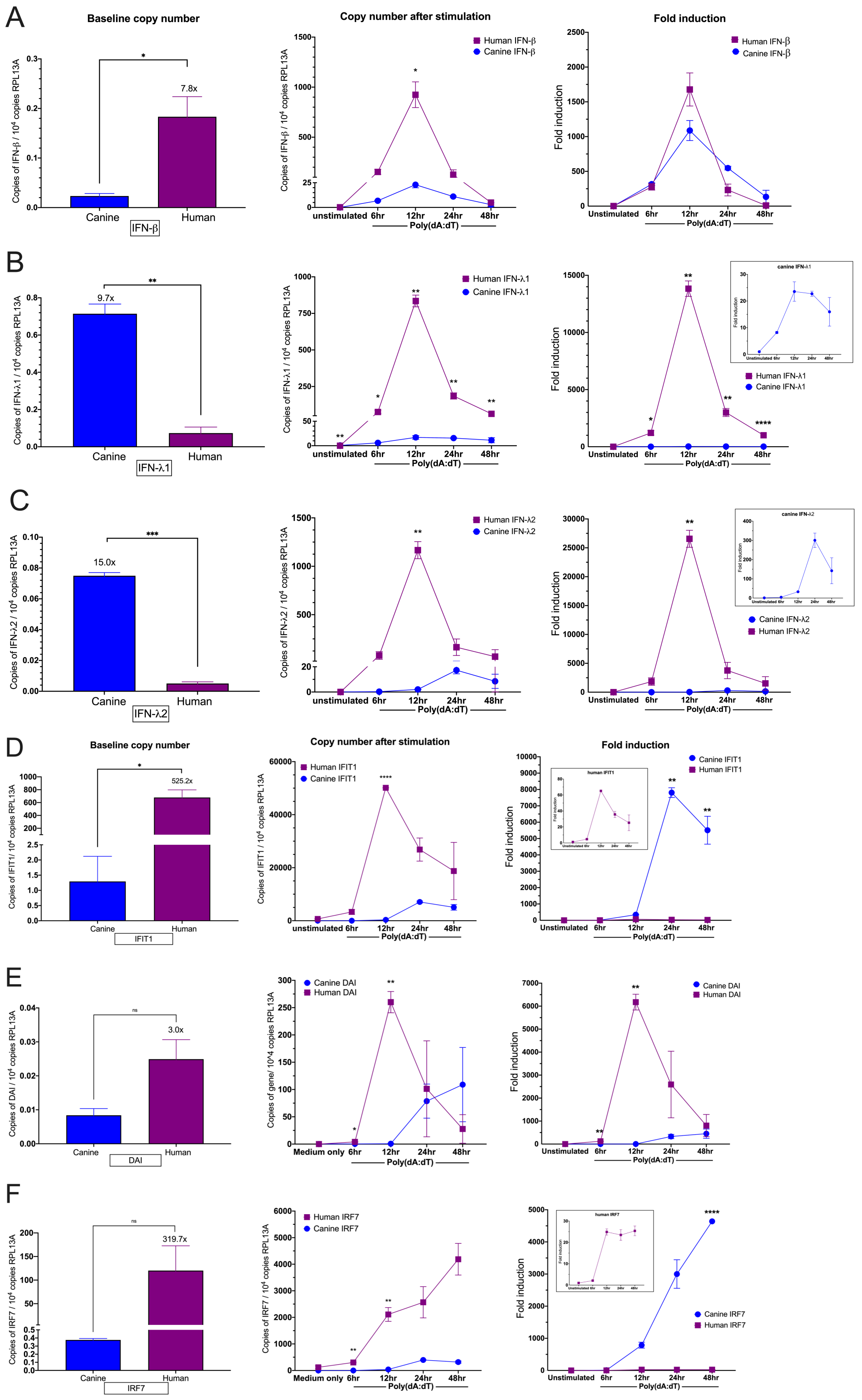

Supplemental Figure 2. Absolute copy number and fold expression of IFNs and ISGs in human and canine keratinocytes at baseline and after Poly(dA:dT) stimulation. Basal copy number, induced copy number after Poly(dA:dT) stimulation, and relative fold induction after Poly(dA:dT) stimulation of IFN-β (**A**), IFN-λ1 (**B**), IFN-λ2 (**C**), IFIT1 (**D**), DAI (**E**) and IRF7 (**F**) in human NHEKs and canine CPEKs are shown. Gene copy number was normalized to 10^4 copies of RPL13A, and relative fold induction is shown as ΔΔCt of mRNA expression over unstimulated control (set to 1), and normalized to RPL13A. Data is presented as mean ± SD of one representative experiment of two. Asterisks (*) indicate a significant difference (*p*<0.05) in expression between NHEKs and CPEKs, where *p < 0.05; **p < 0.01; ***p < 0.001; ****p < 0.0001.

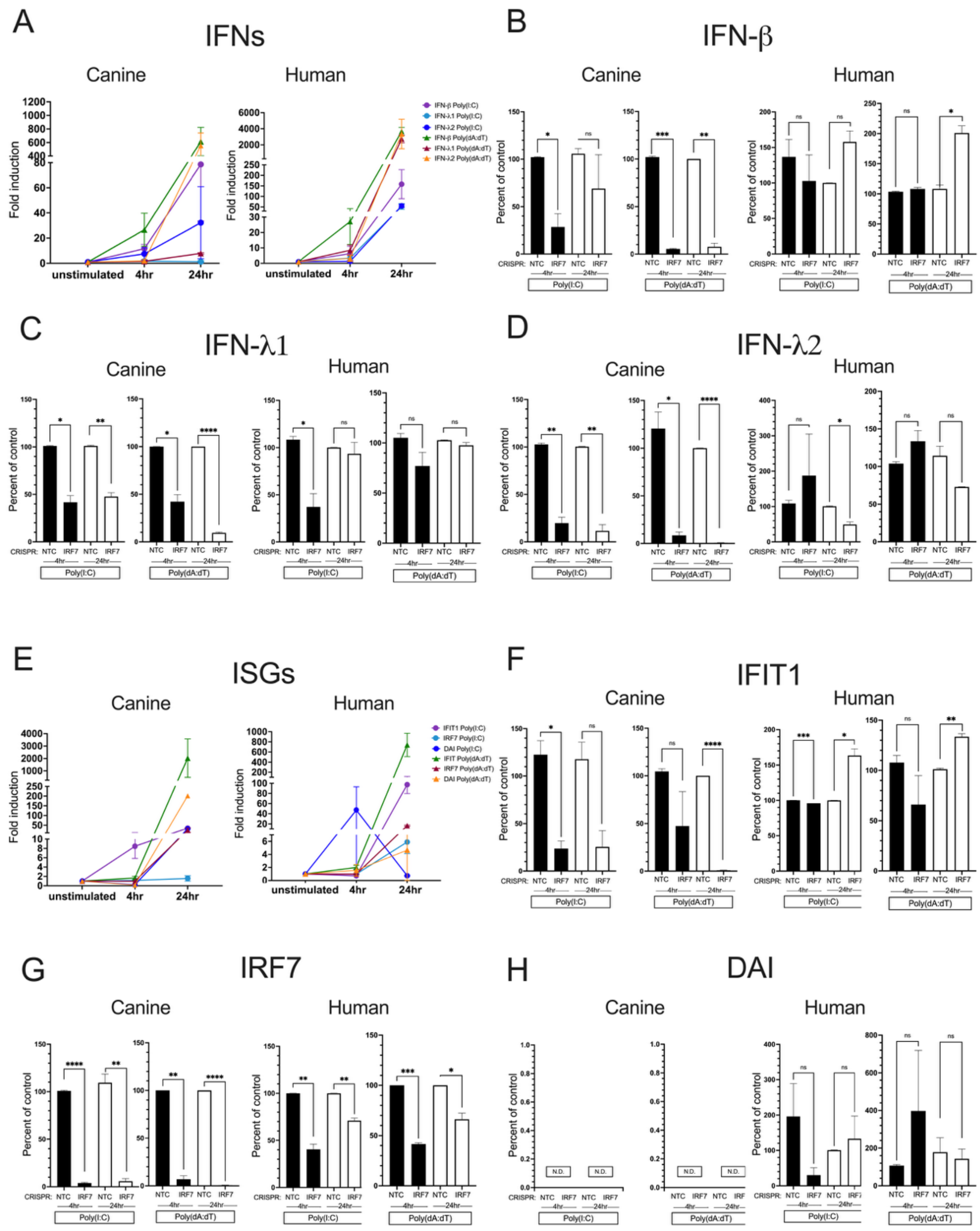

Supplemental Figure 3: Induced expression of IFNs and ISGs in human cells HEK-293 and canine epithelial cells MDCKs with knockdown (KD) of IRF7. Non-targeting control (NTC) cells and IRF7 KD cells were assessed by quantitative RT-PCR for IFN and ISG expression levels 4 and 24 hours post stimulation with Poly(I:C) or Poly(dA:dT). Fold induction (ΔΔCt) of IFN **(A)** or ISG **(E)** mRNA expression in NTC MDCKs or HEK-293s. Fold induction is graphed as expression in NTC cells over the unstimulated control (set to 1), and normalized to the reference gene, RPL13A. Expression of IFN-β **(B)**, IFN-λ1 (**C**), IFN-λ2 (**D**), IFIT1 **(F)**, IRF7 (**G**), and DAI (**H**) in IRF7 KD MDCKs and HEK-293s after Poly(I:C) or Poly(dA:dT) stimulation is graphed as the percent compared to NTC cells normalized to the reference gene RPL13A. Data represents the mean of 3 independent experiments performed in duplicate, ± SEM. Asterisks (*) indicate a significant difference (*p*<0.05) in expression between NTC control and IRF7 KD cells, where *p < 0.05; **p < 0.01; ***p < 0.001; ****p < 0.0001. ns= not significant. N.D. = not detected.
